# Characterization of *Drosophila ATPsynC* mutants as a new model of mitochondrial ATP synthase disorders

**DOI:** 10.1371/journal.pone.0201811

**Published:** 2018-08-10

**Authors:** Domenica Lovero, Luca Giordano, René Massimiliano Marsano, Alvaro Sanchez-Martinez, Hadi Boukhatmi, Maik Drechsler, Marta Oliva, Alexander J. Whitworth, Damiano Porcelli, Corrado Caggese

**Affiliations:** 1 Dipartimento di Biologia, Università degli Studi di Bari “Aldo Moro”, Bari, Italy; 2 Medical Research Council Mitochondrial Biology Unit, Cambridge Biomedical Campus, Cambridge, United Kingdom; 3 Department of Physiology, Development and Neuroscience, University of Cambridge, Cambridge, United Kingdom; 4 Department of Zoology, University of Cambridge, Cambridge, United Kingdom; Biomedical Sciences Research Center Alexander Fleming, GREECE

## Abstract

Mitochondrial disorders associated with genetic defects of the ATP synthase are among the most deleterious diseases of the neuromuscular system that primarily manifest in newborns. Nevertheless, the number of established animal models for the elucidation of the molecular mechanisms behind such pathologies is limited. In this paper, we target the *Drosophila melanogaster* gene encoding for the ATP synthase subunit c, *ATPsynC*, in order to create a fruit fly model for investigating defects in mitochondrial bioenergetics and to better understand the comprehensive pathological spectrum associated with mitochondrial ATP synthase dysfunctions. Using P-element and EMS mutagenesis, we isolated a set of mutations showing a wide range of effects, from larval lethality to complex pleiotropic phenotypes encompassing developmental delay, early adult lethality, hypoactivity, sterility, hypofertility, aberrant male courtship behavior, locomotor defects and aberrant gonadogenesis. *ATPsynC* mutations impair ATP synthesis and mitochondrial morphology, and represent a powerful toolkit for the screening of genetic modifiers that can lead to potential therapeutic solutions. Furthermore, the molecular characterization of *ATPsynC* mutations allowed us to better understand the genetics of the *ATPsynC* locus and to define three broad pathological consequences of mutations affecting the mitochondrial ATP synthase functionality in *Drosophila*: i) pre-adult lethality; ii) multi-trait pathology accompanied by early adult lethality; iii) multi-trait adult pathology. We finally predict plausible parallelisms with genetic defects of mitochondrial ATP synthase in humans.

## Introduction

The mitochondrial oxidative phosphorylation (OXPHOS) system provides the bulk of cellular energy in all eukaryotic cells. This essential biological process is under the dual control of nuclear and mitochondrial genomes [1].

Consequently, inborn genetic defects of the OXPHOS system cause severe diseases, characterized by early-onset and highly variable disorders such as neuromyopathies and cardiomyopathies, with limited therapies yet available for the treatment of patients [2].

A pivotal role in mitochondrial energy production is played by the ATP synthase (also known as F_1_F_O_-ATPase or Complex V), which catalyses the synthesis of adenosine triphosphate (ATP) from adenosine diphosphate (ADP) and inorganic phosphate (Pi) using the transmembrane proton-motive force generated by the respiratory chain complexes [3].

**Fig 1:**
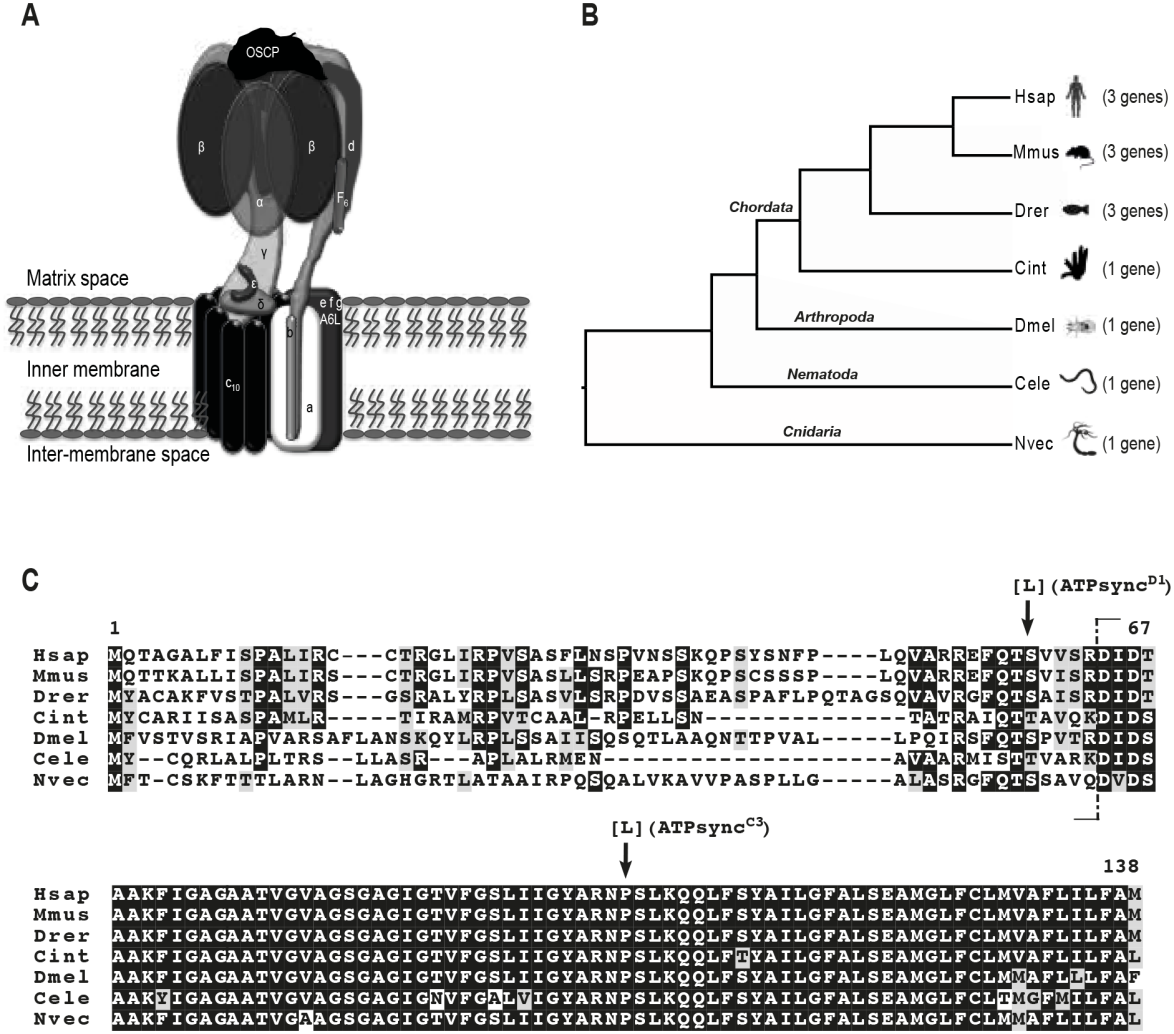
The mitochondrial ATP synthase and its subunit c. (A) Structural organization of the mature mitochondrial ATP synthase. (B-C) Number of genes and protein alignment of the mitochondrial ATP synthase subunits c from representative metazoan species (see text for details). Hsap = *Homo sapiens*; Mmus = *Mus musculus*; Drer = *Danio rerio*; Cint = *Ciona intestinalis*; Dmel = *Drosophila melanogaster*; Cele = *Caenorhabditis elegans*; Nvec = *Nematostella vectensis*. (C) The dashed vertical line separates the N-terminal mitochondrial targeting peptide from the mature subunit c; arrows point to amino acid substitutions, due to EMS-induced mutations, in the coding sequence of *ATPsynC*^*C3*^ and *ATPsynC*^*D1*^ alleles.

In mammals, the ATP synthase is composed of at least 16 different subunits organized into two major functional domains connected by two stalks [4] (Fig 1A): the F_1_ domain, projected into the mitochondrial matrix, and the F_O_ domain, embedded in the mitochondrial inner membrane. The matrix F_1_ section is composed of a *α*_*3*_*β*_*3*_ spherical structure and by subunits *γ*, *δ* and *ε*, which form the central stalk and connect F_1_ to F_O_. The latter consists of a ring of eight to ten subunits c (c-ring) plus subunits a, e, f, g, A6L and b. b subunit extends into the matrix and assembles with subunits d, F_6_ and OSCP to form the peripheral stalk connecting the F_O_ part to F_1_. Subunit IF_1_ binds to the F_1_ domain at low pH and prevents the enzyme to switch from ATP synthesis to ATP hydrolysis.

While subunits a and A6L are encoded by the mitochondrial genome (*ATPase6* and *ATPase8* genes, respectively), all the other components of the ATP synthase originate from the expression of nuclear genes. The assembly of ATP synthase complex from single subunits requires the assistance of several chaperones, in a process still not completely understood at the molecular level [5].

Numerous maternally-inherited mutations of the mitochondrial DNA-encoded subunits a and A6L have been identified in addition to single mutations of nuclear genes encoding structural subunits *α* and *ε* (*ATP5A1* and *ATP5E* genes, respectively [6, 7]), and the assembly factor Atp12p (*ATPAF2* gene, [8]). Furthermore, the autosomal *TMEM70* gene, encoding for an ancillary factor of the ATP synthase, has proven to be a mutational hot-spot [9, 10].

Most of these mutations result in severe neuromuscular disorders since they generate perturbations in the correct assembly and/or function of the mitochondrial ATP synthase (for a recent review see [11]). Moreover, the frequency of such genetic defects may be underestimated, as reported earlier [12].

In spite of that, nowadays, very few animal models exist to understand the molecular and pathogenic mechanisms of ATP synthase-related diseases and to help the quest for new and effective therapeutic solutions [13–16].

Here we provide a new animal model as powerful tool for studying mitochondrial ATP synthase defects, obtained by a detailed mutational analysis of the *D*. *melanogaster* nuclear gene *ATPsynC*, encoding for the F_O_ subunit c.

In metazoans, one or more genes encode for the mitochondrial ATP synthase subunit c (Fig 1B), which is initially synthesized as a precursor polypeptide, containing an N-terminal mitochondrial targeting peptide, and then cleaved to produce a short functional protein of 75 amino acids. MitoFates [17] and multiple alignment analyses, can be easily used to predict the cleavage site, showing that the first ultra-conserved residue is located in position 64 of the alignment, while the leader peptide is poorly conserved among the aligned species (Fig 1C). The mature subunit c shows an exceptionally high degree of conservation throughout evolution (Fig 1C), due to specific constraints hinging on its high hydrophobicity and the ability to form a functional c-ring within the F_O_ complex [18]. In fact, in species possessing multiple genes for the subunit c, the encoded mature protein remains identical [19].

In this study, we isolated and characterized a large set of *ATPsynC* mutations affecting either its coding or 5’UTR sequences. These mutations result in severe lethal or highly pleiotropic phenotypes with different penetrance and onset during *Drosophila* development. We demonstrate that *ATPsynC* is essential for viability, cellular ATP synthesis and proper mitochondrial morphology depend on its function.

Furthermore, we attempted to integrate our phenotypic and molecular data into a model, which predicts three defined, broad pathological consequences of ATP synthase deficiencies in *Drosophila*.

## Materials and methods

### Fly strains and isolation of mutants

#### P-element-mediated hybrid dysgenesis

Oregon-R (OR) wild-type flies were used as controls in all experiments. The stocks carrying a P-element insertion in the 100B7 region, *P{SUPorP}CG1746*^*KG01914*^ and *P{EP}CG1746*^*EP(3)3264*^, the *Df(3R)Exel6218* deletion, balancer chromosomes and the other stocks used were provided by the Bloomington Stock Center. Flies were raised on standard cornmeal/yeast/agar medium at 18°C, and all crosses were made at 25°C, whereas developmental assays were performed at 22°C.

In this work, *P{SUPorP}CG1746*^*KG01914*^ *and P{EP}CG1746*^*EP(3)3264*^ insertions are renamed *ATPsynC*^*1*^ and *ATPsynC*^*2*^, respectively. An independent recessive lethal mutation is present on the third chromosome of both stocks that carries the original *ATPsynC*^*1*^ and *ATPsynC*^*2*^ insertions. Heterozygous flies *ATPsynC*^*1*^*/ATPsynC*^*+*^ and *ATPsynC*^*2*^*/ATPsynC*^*+*^ have wild-type phenotype and are comparable to control Oregon-R flies. To perform a secondary P-element mutagenesis, the *ATPsynC*^*1*^*-P{SUPorP}* and *ATPsynC*^*2*^*-P{EP}* elements were mobilized by providing a Δ2–3 external source of transposase [20]. To generate and identify events mobilizing the P-element in the *ATPsynC*^*1*^ allele, *y;ATPsynC*^*1*^*/TM3*,*Ser*,*Sb* females were crossed to *y*^*+*^ *w*^*+*^*;SbΔ2-3(99B)/TM6* males, which carry a stable source of Δ2–3 transposase integrated on the third chromosome. Male progeny showing a variegated eye pigmentation (genotype *y*; *ATPsynC*^*1*^, *y*^*+*^*/SbΔ2–3*) was collected and crossed to *y; ATPsynC*^*1*^*/TM3*,*Ser*,*Sb* females. *yellow* males of the progeny were individually crossed to *y; ATPsynC*^*1*^*/TM3*,*Ser*,*Sb*. Yellow-body *Sb (stubble)* lines were established and propagated, and the selected revertant alleles were tested for lethality and fertility in trans-heterozygosis with either the *ATPsynC*^*1*^ and *ATPsynC*^*2*^ insertion or with the *Df(3R)Exel6218* deletion. The same protocol was used to recover revertant alleles from the *ATPsynC*^*2*^ insertion line, but in this case we used *w* (white eye colour) as a selectable marker. To define the lethality stage associated with lethal *ATPsynC* alleles, the *TM3*,*Ser*,*Sb* balancer chromosome was replaced by *TM6*,*Tb*. Isogenization of the genetic background was achieved by repeated backcrosses against a laboratory strain carrying balancer chromosomes.

#### Ethyl methane sulphonate (EMS) mutagenesis

Mutagenesis was performed according to standard protocols [21]. OR males were starved in empty vials at room temperature for 10 h prior to the treatment. After starvation, 1400 males were transferred to bottles containing a paper tissue soaked in 5 mL of a 1% sucrose solution containing 25 mM EMS (Sigma-Aldrich Co. LLC.), where they were kept for 24 h. Afterwards, they were divided into four groups containing medium, together with 3000 *ATPsynC*^*2*^*/TM6B*,*Tb* virgin females (F0 cross) and maintained at 25°C for two days. Subsequently, all the F0 males were discarded to avoid mutation clustering. F0 females were then moved every two days in new bottles up to a total of three bottles per group. 7000 F1 white-eyed *Tb+* males were collected and crossed individually to three *ATPsynC*^*2*^*/TM6B*,*Tb* virgin females. Individual crosses where the *ATPsynC*^*2*^ insertion was not complemented were taken as putative *ATPsynC* mutant alleles, which were then re-tested against *ATPsynC*^*1*^ and *Df(3R)Exel6218* null alleles and against the wild-type Oregon-R flies. Isogenization was performed as described above.

### RNA and DNA techniques

Total RNA for gene expression analysis was obtained from 0.5 to 1g samples of flies of Oregon-R and *ATPsynC*^*1rev*^ lines at various developmental stages (larvae, pupae and adult flies). Male and female individuals contributed equally to each sample. Total RNA was isolated using the RNeasy mini kit (QIAGEN) according to manufacturer’s instruction. RNA was quantified using a Nanodrop spectrophotometer (Thermo Scientific) and 1μg of RNA was reverse transcribed using the QuantiTect Reverse Transcription kit (QIAGEN) according to the protocol supplied by the manufacturer. Quantitative real-time PCR was performed using the ABI PRISM 7300 Real-Time PCR System (Applied BioSystems). Reactions were set up in 10 μl total volume as follows: 5 μl of 2X Power SYBR Green PCR Master Mix (Applied Biosystems), 2 pmol forward and reverse primers, and 2 μl of a 1:10 dilution of the cDNA synthesis reaction as a template. All reactions were performed at least in triplicate. Specificity and identity of the amplification products were confirmed by analysis of the dissociation curves. ΔCt values were obtained using the *rp49 (Rpl32)* gene, which encodes a constitutively expressed ribosomal protein, as the internal control. Primers used for real-time amplification were qRT_ATPsynC_Up: 5’-GCCGCAACAGTCGGTGTC-3’, qRT_ATPsynC_Lw: 5’-AGGCGAACAGCAGCAGGAA-3’(deriving from the coding sequence of *ATPsynC* gene), qRT_rp49_Up: 5’-CGCAAGCCCAAGGGTATC-3’ and qRT_rp49_Lw: 5’-ATCTCGCCGCAGTAAACG-3’. For the Northern blot anayses, RNA samples (5–10μg) were separated on 1% formaldehyde agarose gel, transferred to a nylon membrane (Hybond-N, GE Healthcare), hybridized and washed using high stringency conditions, and exposed [22]. A 318 bp-long cDNA probe (mapping between positions 97 and 414 of the coding sequence) was obtained from reverse transcription of *ATPsynC* mRNA and labelled at high specific activity with ^32^P[dATP]. A cDNA probe relative to the *rp49* gene was used as a loading control. Standard DNA manipulations were performed as previously described [22]. Genomic DNA from heterozygous mutant flies carrying a single P-element insertion was amplified using oligonucleotide primers derived from the terminal inverted repeat sequence of the P-element (Pterm: 5’-CGGGACCACCTTATGTTATTT-3’) and a primer (ATPsynCUp2: 5’-TTGGGTTCTCAACCAAAATG-3’) corresponding to position 40 upstream of the *ATPsynC* TSS (Transcription Start Site). The primers used for PCR analysis of excision events were the Pterm and ATPsynCUp2 described earlier and a genomic primer located at position +683 from the TSS (ATPsynCLw2: 5’-GCGAAATTTCACACGATTCT-3’). Amplification products were either directly sequenced or cloned into a pGEM-T vector (Promega) and then sequenced. Genomic DNA from heterozygous mutant flies deriving from EMS-mutagenesis was amplified using three pairs of primers designed along the three exon regions. The gel-purified amplification products were sequenced.

### Protein purification and analysis by SDS—PAGE and western immunoblot

Adults and larvae were directly homogenized in SDS gel-2X Laemmli sample buffer (65.8 mM Tris-HCl, pH6.8, 2.1% SDS, 26.3% (w/v) glycerol, 0.01% bromophenol blue, Bio-Rad) with the addition of β-mercaptoethanol and heated at 95°C for 5 min. Homogenates were centrifuged at 13000X g, and an aliquot of the soluble protein fractions were electrophoresed on 4,5–12% SDS-polyacrylamide gel in TGS buffer (25 mM Tris, 192 mM Glycine, 0,1% SDS) and then electro-transferred over night to a Nitrocellulose membrane (Hybond-C-Extra, GE Healthcare) in Transfer Buffer (25 mM Tris, 192 mM Glycine, 20% methanol).

The Nitrocellulose membrane was blocked for 1 h in TBST (Tris-HCl 1 M, pH 8; 150 mM NaCl, 0.1% Tween 20) supplemented with 5% non-fat milk (Bio-Rad). After blocking, proteins were probed with a polyclonal antibody raised in rabbit against the amino-terminal DIDSAAKFIGA epitope of mature ATPsynC (diluted 1:2000) (a gift from Dr. Elizabeth F. Neufeld), and with a polyclonal antibody raised in mouse against Porin (diluted 1:2000), used as a control. Detection was carried out using a 1:5000 dilution of anti-rabbit and anti—mouse secondary antibodies conjugated to AP. We utilized Bio-Rad’s AP-based colorimetric kits with 5-bromo-4-chloro-3-indolyl phosphate/Nitroblue Tetrazolium (BCIP/NBT) reagents, which react with the alkaline phosphatase to form a colour precipitate for detection.

### Measurement of intracellular ATP levels

ATP levels were determined by the luciferin/luciferase assay with the aid of Glomax 20/20 luminometer (Promega). Adult flies wild-type (OR) and trans-heterozygotes for the *ATPsynC*^*1rev*^ alleles were collected in Eppendorf tubes and then homogenized in PBS using a potter homogenizer. An aliquot was incubated on ice with 12% perchloric acid for 1 min and then neutralized with a solution of 3M K_2_CO_3_ and 2M Tris. After centrifugation at 13000 rpm for 1 min, ATP content was measured at 25 °C, using the ATP Bioluminescence Assay kit (Sigma-Aldrich Co. LLC.), according to manufacturer’s instruction. The picomoles of ATP were calculated by comparison with an appropriate internal ATP standard (25 pmol), and normalized to total protein content, assessed using Bradford’s method [23].

### Locomotory behaviour assays

Behavioural assays were performed as reported in [24] and [25]. Twenty groups of 10 flies (5 females and 5 males, equally aged) were collected from populations of different genotypes: Oregon-R (wild-type), *ATPsynC*^*1rev17*.*1*^*/ATPsynC*^*2*^, *ATPsynC*^*1rev18*.*3*^*/ATPsynC*^*2*^, *ATPsynC*^*1rev5*.*2*^*/ATPsynC*^*2*^ and *ATPsynC*^*1rev24*.*2*^*/ATPsynC*^*2*^. Flies were maintained at 25° and tested within 96 hours. Each sample was used for the running, climbing and flight tests, in this sequence. Behavioural tests were performed after at least 6 hours of recovery from anaesthesia.

Running test: groups of 10 flies (n = 14) were placed in a 10-ml glass pipette (20 cm of length) darkened for its entire length but illuminated at one end by a fiber optic lamp.

To start the test, the flies were knocked down to the darkened end of the pipette, which was then returned to the horizontal position. The time required for the first six flies of the sample to reach the lighted end of the pipette was recorded. Four trials were completed for each sample. The recorded times were then transformed in “performance coefficients” according to the following scale: (1) ⩽30 s; (2) 31–60 s; (3) 61–90 s; (4) 91–120 s; (5) 121–150 s; (6) 151–180 s; (7) 181–210 s; (8) 211–240 s; (9) 241–270 s; (10) 271–300 s; (11) ⩾300 s. Scores for each sample were then averaged for statistical analysis using Anova one-way test followed by the Bonferroni post-hoc test.

Climbing test: groups of 10 flies (n = 12) were placed in a 250-ml glass graduated cylinder illuminated at the top by a fiber optic lamp. To start the test, the flies were knocked down to the bottom of the cylinder and the time required for half the sample to reach the 150 ml line on the cylinder wall (17.5 cm) was recorded. Four trials were completed for each sample. Times for each sample were averaged for statistical analysis using Anova one-way test followed by the Bonferroni post-hoc test.

Flight assay: flies from each sample were dropped into a 500-ml graduated cylinder through a funnel whose end reached the 500-ml mark. The internal wall of the cylinder was coated with paraffin oil, causing flies to become stuck when striking the paraffin film. The vertical distribution of the flies over the length of the cylinder reflects the flight ability of the individuals because stronger fliers become stuck to the wall near the top of the container, weaker fliers near the bottom. The distance from the top of the cylinder at which each fly was stuck was recorded and transformed in a “performance coefficient” according to the following scale: (1) <3 cm; (2) 3–6 cm; (3) 6–9 cm; (4) 9–12 cm; (5) 12–15 cm; (6) 15–18 cm; (7) 18–21 cm; (8) 21–24 cm; (9) 24–27 cm; (10) 27–30 cm. Scores for each sample were averaged for statistical analysis using Anova one-way test followed by the Bonferroni post-hoc test.

### Mating and fertility assay

Initially, virgin males and females of each genotype were collected and transferred separately into new vials containing standard food medium and live yeast and left develop for 4–5 days until they were sexually mature. A virgin female was placed with a virgin male in a fresh food vial and the start and end of copulation was recorded. The male was then removed and the female was allowed to lay eggs for two days in the same vials before being moved to a fresh vial every two days for a total of three vials per female. The egg laid and the number of the adults developed were scored.

### Transmission electron microscopy (TEM)

Thoraces were prepared from 5-day old adult flies and treated as previously described [26]. Briefly, thoraces were dissected in PBS-Tween 0.5% and fixed overnight at 4°C in half strength Karnovsky’s fixative. After washing in 0.1M sodium cacodylate, samples were post-fix stained in 2% Osmium Tetroxide and 0.2M cacodylate 1:1 for 1 hour. After several dehydration steps in an ethanol series the tissue was placed in PO/EPON resin 50:50 for 4 hours and transferred to fresh EPON resin for 3 days replacing it every 24 hours. Specimens were oriented and placed in the oven at 60°C for minimum 48 hours. Ultra-thin sections taken in a Reichert-Jung Ultracut E ultramicrotome were examined using a TEM (FEI Tecnai G2 Spirit 120KV).

### Immunofluorescence microscopy

Adult indirect flight muscles were prepared and stained as described in [27]. The following primary antibodies were used: Goat anti-GFP (1:200, Abcam, ab6673), Rabbit anti-Mef2 (1:200, a gift from Eileen Furlong, Heidelberg, Germany), Alexa-conjugated Phalloidin (1:200, Thermo fisher). Samples were imaged on Leica TCS SP8 microscope (CAIC, University of Cambridge) at 20X and 40X magnification and 1024/1024 pixel resolution. Images were processed with Image J.

## Results

### A single locus encodes for the subunit c of the mitochondrial ATP synthase in *Drosophila*

In *D*. *melanogaster*, the subunit c of the mitochondrial ATP synthase is encoded by a single gene, *ATPsynC*, located close to the telomeric region of chromosome 3R, within the polytene band 100B7 [28].

**Fig 2:**
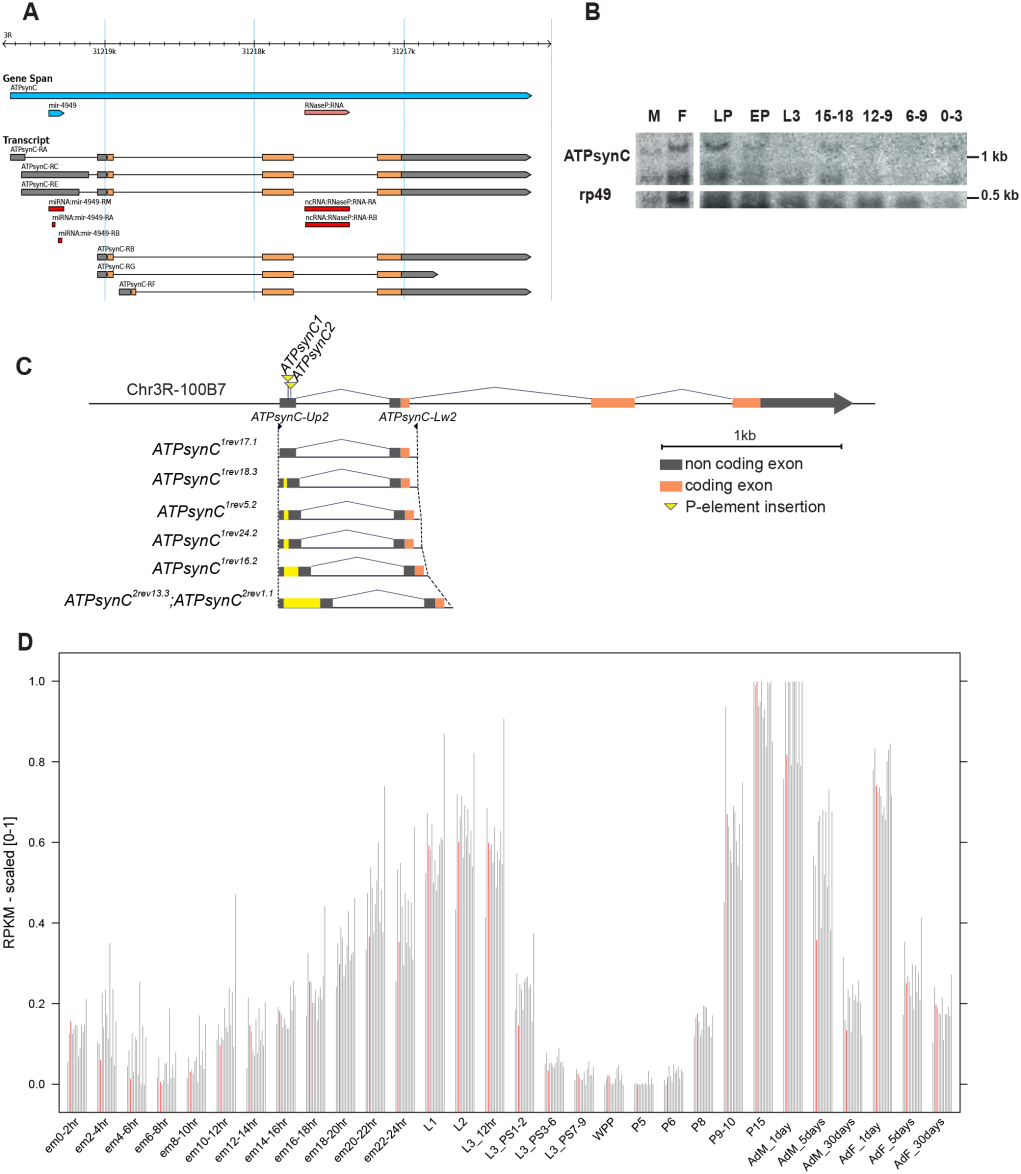
The *Drosophila melanogaster ATPsynC* gene. (A) FlyBase annotation of *ATPsynC* (FBgn0039830). Untranslated regions are depicted in grey; CDSs are depicted in light orange. Non-coding RNAs also transcribed from the *ATPsynC* locus are shown in red. (B) Northern blot analysis of *ATPsynC* expression during *Drosophila* development, compared to the housekeeping gene *rp49*. M = adult male (mixed age); F = adult female (mixed age); LP = late pupal stage; EP = early pupal stage; L3 = third instar larval stage; 0–3, 6–9, 12–9 and 15–18 correspond to hours of embryonic development at 25°C. (C) Schematic representation of *ATPsynC* P-element insertions and the alleles derived after transposon remobilization. (D) Expression analysis of the 13 nuclear genes encoding for subunits of the mitochondrial ATP synthase (CG1746, CG3612, CG11154, CG7610, CG2968, CG9032, CG8189, CG6030, CG3321, CG4692, CG6105, CG4412, CG4307). Expression values derive from the modENCODE project and are reported as scaled [0–1] RPKM (Reads Per Kilobase of transcript per Million mapped reads); red bars indicate *ATPsynC* values.

According to the current gene model annotations (Fig 2A, see also FlyBase ID FBgn0039830), the *ATPsynC* locus consists of different transcriptional units that can produce up to six different mRNA isoforms transcribed from 4 different TSSs. While CG1746-RA, CG1746-RB, CG1746-RC, CG1746-RG and CG1746-RE isoforms give rise to the same protein (MITOPROT mitochondrial location probability 0,9189), CG1746-RF results in a protein with a different amino-terminal sequence (MITOPROT mitochondrial location probability 0,32). However, all the alternative mRNAs probably encode for the same putative, mature polypeptide. Our Northern blot analyses show that *ATPsynC* produces two hybridization bands. The higher molecular weight band is likely related to the RA, RB and RF isoforms (respectively 1439 bp, 1347 bp and 1350 bp long, Fig 2B), while the lower molecular weight band could be related to the RG isoform (722 bp long, Fig 2B). Therefore, it can be assumed that the above mentioned isoforms constitute the bulk of mitochondrial ATP synthase subunit c expression throughout *D*. *melanogaster* development, while the expression of RC and RE isoforms is under the limit of detection of the Northern blot technique. However, as discussed later in this work, we provide evidence that the RA isoform could represent the main transcriptional isoform.

This main transcriptional unit, the only one we will refer to hereafter, consists of four exons and three introns (Fig 2C). The first and third introns contain independent non-coding RNAs *mir-4949* and *Ribonuclease P RNA (RnaseP*:*RNA)*, respectively [28, 29] (see Fig 2A).

Detailed temporal expression data from the modENCODE project show that *ATPsynC* is highly and constantly expressed during *Drosophila* development with peaks of expression from first to early third larval stages (L1, L2, L3 12 hrs) and during the transition from pupal to adult stage (P9-P15, AdM and AdF 1day) (Panel A in S1 Fig and Fig 2D). Furthermore, *ATPsynC* and the other nuclear genes encoding for the remaining subunits of the mitochondrial ATP synthase exhibit an extremely tight co-expression throughout development, as shown here for the first time for a metazoan species (Fig 2D). While these data derive from RNA-seq experiments, our qRT-PCR analyses found that 5 days old, wild-type Oregon-R (OR) adult males express roughly a double amount of *ATPsynC* mRNA compared to their female counterparts (Panel B in S1 Fig), a trend common to other ATP synthase nuclear genes (Fig 2D).

The precursor polypeptide encoded by *ATPsynC* is 138 amino acids long and consists of a putative mitochondrial import sequence of 63 residues, which is cleaved off in order to produce a putative mature, functional protein of 75 amino acids (Fig 1C). As for many mitochondrial proteins encoded by nuclear genes, the N-terminal signal peptide shows an opposite degree of evolutionary constraint than the remaining mature portion of the ATP synthase subunit c, which has barely changed throughout metazoan evolution (Fig 1B).

To investigate the consequences of deleterious *ATPsynC* mutations, we carried out an extensive mutagenesis analysis, by implementing traditional P-element and ethyl methanesulfonate (EMS) techniques leading to the identification of a pool of very informative mutations displaying a wide range of phenotypes.

### P-element mediated mutagenesis of *ATPsynC*

We used two distinct *D*. *melanogaster* lines carrying engineered P-elements, inserted in the first exon of *ATPsynC* gene, to generate a large set of new *ATPsynC* mutant alleles by transposon remobilization (Fig 2C, see Material and methods).

By using standard PCR and Sanger sequencing, we precisely mapped these two P-insertions, *P{SUPor-P}KG01914* and *P{EP}EP3264* (hereafter denominated as alleles *ATPsynC*^*1*^ and *ATPsynC*^*2*^), at 67 and 84 nucleotides downstream the annotated TSS of *ATPsynC*, respectively.

Interestingly, these insertions affect gene regions that are highly conserved among *Drosophila* species (S2 Fig) and that may play important roles during both the transcription of *ATPsynC* and/or the post-transcriptional processing of its mRNAs.

In fact, both *ATPsynC*^*1*^ and *ATPsynC*^*2*^ show a recessive, severe lethal phenotype when tested in the trans-heterozygous combination *ATPsynC*^*1*^*/ATPsynC*^*2*^. Because independent lethal mutations are present on the third chromosome of both stocks carrying *ATPsynC*^*1*^ and *ATPsynC*^*2*^ (data not shown), phenotypic analyses concerning these alleles, and those derived from re-mobilization, were carried out on trans-heterozygotes (see Table 1). Moreover, either of the *ATPsynC* insertions in trans to a deficiency mutation which encompasses the *ATPsynC* locus, *Df(3R)Exel6218* [30], show the same lethal phenotype (Table 1).

**Table 1:** *ATPsynC* alleles and phenotypes.

Allele	Phenotype	Mutational Event	Levels of Protein Expression, compared to wild-type (±SD)
*ATPsynC*^*1*^	Lethal	P{SUPor-P} 5’ UTR insertion	0%
*ATPsynC*^*2*^	Lethal	P{EP} 5’ URT insertion	0%
*ATPsynC*^*1rev16*.*2*^	Lethal	75 bp P{SUPor-P} insertion	6.9% (±4.3)
*ATPsynC*^*2rev1*.*1*^	Lethal	202 bp P{EP} insertion	NA
*ATPsynC*^*2rev13*.*3*^	Lethal	202 bp P{EP} insertion	NA
*ATPsynC*^*D1*^	Lethal	missense S59L	0%
*ATPsynC*^*C3*^	Lethal	missense P103L	0%
*ATPsynC*^*1rev18*.*3*^	*Pleiotropic*	13 bp P{SUPor-P} insertion	60.6% (±12.9)
*ATPsynC*^*1rev5*.*2*^	*Pleiotropic*	36 bp P{SUPor-P} insertion	44.3% (±21.2)
*ATPsynC*^*1rev24*.*2*^	*Pleiotropic*	38 bp P{SUPor-P} insertion	48.7% (±23.7)
*ATPsynC*^*1rev25*.*3*^	Lethal	Large P{SUPor-P} insertion	NA
*ATPsynC*^*1rev42*.*2*^	Lethal	Large P{SUPor-P} insertion	NA
*ATPsynC*^*2rev7*.*2*^	Lethal	Large P{EP} insertion/deletion	NA
*ATPsynC*^*2rev10*.*2*^	Lethal	Locus deletion	NA
*ATPsynC*^*1rev17*.*1*^	Wild-type	Perfect excision	118% (±37)

(NA: not analysed)

**Fig 3:**
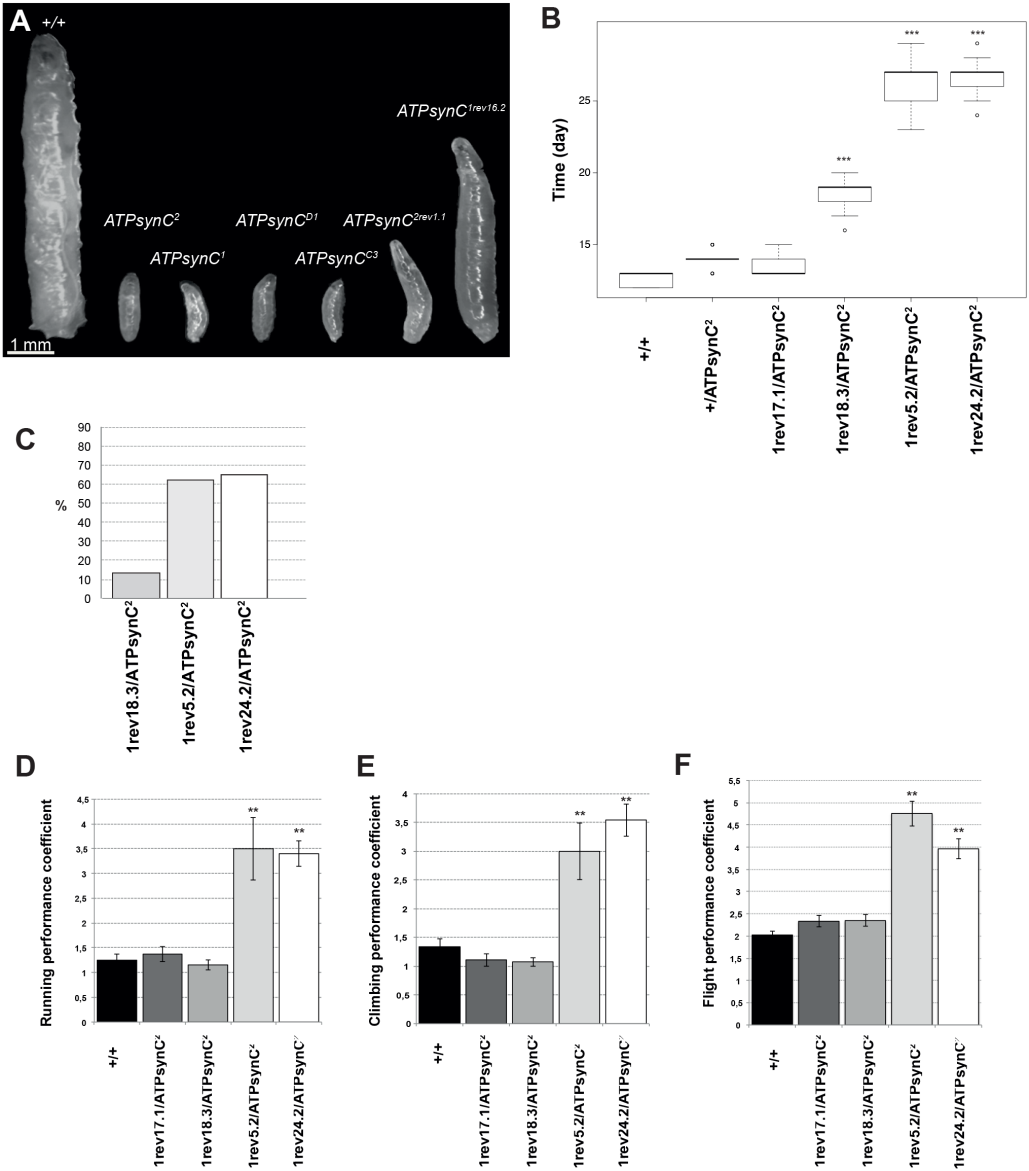
Phenotypic characterization of *ATPsynC* mutant alleles. (A) Maximal larval development of lethal mutations of *ATPsynC*. (B) Generation time (from egg to adult) of representative revertant alleles produced by excision of the P-element insertion in *ATPsynC*^*2*^. (C) Percentages of early adult lethality of individuals trans-heterozygotes for the *ATPsynC* pleiotropic mutations characterized in this work. (D-F) Running, climbing and flying performances of representative revertant alleles produced by excision of the P-element insertion in *ATPsynC*^*2*^. Higher performance coefficients indicate worse locomotor skills (see Materials and methods section). Statistical significance codes (comparison to wild-type): **P < 0.01. +/+ = Oregon-R flies.

These lethal phenotypes are associated with a developmental arrest at the first-instar larval stage, which is prolonged for about ten days when mutant larvae become extremely hypoactive and subsequently die (see Fig 3A).

Dysgenic crosses were performed to re-mobilize the P-elements in *ATPsynC*^*1*^ and *ATPsynC*^*2*^ to produce revertant alleles *ATPsynC*^*1rev*^ and *ATPsynC*^*2rev*^, respectively. We isolated a total of 106 alleles, derived from at least 52 independent excision events. *ATPsynC*^*1rev*^ and *ATPsynC*^*2rev*^ series were tested in trans-heterozygosis against *ATPsynC*^*2*^ and *ATPsynC*^*1*^ respectively and against the *Df(3R)Exel6218* deletion in order to determine their phenotypes. Forty-four *ATPsynC*^*1rev*^ and fifty-two *ATPsynC*^*2rev*^ alleles showed a wild-type phenotype; three *ATPsynC*^*1rev*^ alleles generated flies with a highly pleiotropic phenotype, affecting multiple traits (see below); finally, three *ATPsynC*^*1rev*^ and four *ATPsynC*^*2rev*^ alleles still exhibited a lethal phenotype after the P-element excision event. Representative revertant alleles obtained from *ATPsynC*^*1*^ and from *ATPsynC*^*2*^ are shown in Fig 2C and listed in Table 1.

We performed a detailed PCR analysis and sequencing of the revertant alleles using primers designed on the genomic sequences flanking the original *ATPsynC*^*1*^ and *ATPsynC*^*2*^ insertions and on the P-element terminals. We found that all the revertant alleles having a wild-type phenotype derived from perfect P-element excisions while all those associated with lethal or pleiotropic phenotypes derived from imprecise P-element excision events that yielded new *ATPsynC* allelic variants (Table 1 and Fig 2C).

We observed a strong correlation between the amount of P-element left in *ATPsynC* 5’UTR due to imprecise excision, and the severity of the associated phenotype. Alleles *ATPsynC*^*1rev18*.*3*^, *ATPsynC*^*1rev5*.*2*^ and *ATPsynC*^*1rev24*.*2*^, carrying insertions of 13, 36 and 38 bp respectively, showed pleiotropic effects from moderately (*ATPsynC*^*1rev18*.*3*^) to highly penetrant (*ATPsynC*^*1rev5*.*2*^ and *ATPsynC*^*1rev24*.*2*^). Allele *ATPsynC*^*1rev16*.*2*^, carrying an insertion of 75 bp, produced a lethal phenotype consisting of flies that could not develop beyond the early third-instar larval stage (Fig 3A). Independent but identical insertions of 202 bp in alleles *ATPsynC*^*2rev1*.*1*^ and *ATPsynC*^*2rev13*.*3*^ determined lethality at the second instar larval stage (Fig 3A). Finally, large, uncharacterized amounts of the P-element left in alleles *ATPsynC*^*1rev25*.*3*^ and *ATPsynC*^*1rev42*.*2*^ were responsible for the same lethal phenotype as the original *ATPsynC*^*1*^ insertion.

Two additional *ATPsynC*^*2*^ lethal revertant alleles, instead, presented a diverse type of mutation. In *ATPsynC*^*2rev7*.*2*^, the excision event left a substantial part of the *P{EP}* 3’ portion, but generated an upstream deletion of undefined length, while *ATPsynC*^*2rev10*.*2*^ carried an uncharacterized, large deletion of the genomic region encompassing the *ATPsynC* gene. Both these alleles produced the same lethal phenotype as described for *ATPsynC*^*2*^.

### EMS mutagenesis yields two new *ATPsynC* alleles

In order to obtain mutations affecting the coding sequence (CDS) of *ATPsynC*, we performed a non-complementation screen for EMS-induced alleles that were lethal *in trans* to both *ATPsynC*^*1*^ and *ATPsynC*^*2*^. We tested 7000 flies (see Material and methods) and isolated two mutant alleles, named *ATPsynC*^*D1*^ and *ATPsynC*^*C3*^, which were not able to complement the P-element-induced lethal *ATPsynC* alleles, but showed early lethality as for *ATPsynC*^*1*^ and *ATPsynC*^*2*^ (Fig 3A and Table 1). Sequence analyses of these new lethal alleles revealed the presence of a single missense mutation in both mutants. *ATPsynC*^*D1*^ carried the codon substitution TCG -> TTG resulting in the missense S59L mutation, while *ATPsynC*^*C3*^ carried the codon substitution CCA -> CTA resulting in the missense P103L mutation (Fig 1).

### The pleiotropic phenotypes of *ATPsynC*^1rev18.3^, *ATPsynC*^1rev5.2^ and *ATPsynC*^1rev24.2^ alleles

Beside the isolation and characterization of lethal mutations, which corroborate the assumption that *ATPsynC* is an essential gene in *D*. *melanogaster*, we obtained a deeper understanding of the temporal and spatial-anatomical demand of the mitochondrial ATP synthase subunit c in our model organism by characterizing the pleiotropic phenotypes associated with mutations in alleles *ATPsynC*^*1rev18*.*3*^, *ATPsynC*^*1rev5*.*2*^ and *ATPsynC*^*1rev24*.*2*^. These mutations, in fact, affect many life-history traits, morphological and behavioural traits, although, as we show, *ATPsynC*^*1rev18*.*3*^ manifest less phenotypic penetrance than *ATPsynC*^*1rev5*.*2*^ and *ATPsynC*^*1rev24*.*2*^.

Firstly, these mutations induce a marked developmental delay. While control Oregon-R (+/+), *ATPsynC*^*1rev17*.*1*^*/ATPsynC*^*2*^ (perfect excision/insertion) and +/*ATPsynC*^*2*^ flies have a generation time (from egg to adult) of approximately 13 days at 22°C ±1°C, *ATPsynC*^*1rev18*.*3*^*/ATPsynC*^*2*^, *ATPsynC*^*1rev5*.*2*^*/ATPsynC*^*2*^ and *ATPsynC*^*1rev24*.*2*^*/ATPsynC*^*2*^ trans-heterozygotes complete their development in 19, 27 and 26 days on average, respectively (P < 0.001; Fig 3B). Upon hatching from the puparium, a proportion of the same mutant flies manifests marked hypoactivity and dies quickly by either failing to exit the pupal case or collapsing on the medium immediately. This mortality rate, which mainly affects males (data not shown), is 11.6% for *ATPsynC*^*1rev18*.*3*^*/ATPsynC*^*2*^ adults and increases up to 62.3% for *ATPsynC*^*1rev5*.*2*^*/ATPsynC*^*2*^ and 64.3% for *ATPsynC*^*1rev24*.*2*^*/ATPsynC*^*2*^ individuals (Fig 3C). Survivor adult flies, principally those with genotypes *ATPsynC*^*1rev5*.*2*^*/ATPsynC*^*2*^ and *ATPsynC*^*1rev24*.*2*^*/ATPsynC*^*2*^, also show marked hypoactivity.

We investigated locomotory defects caused by these alleles using three standard behavioural assays to analyse running, climbing and flying abilities of mutant flies (see Material and methods for details, [25]). For all the three assays, *ATPsynC*^*1rev5*.*2*^*/ATPsynC*^*2*^ and *ATPsynC*^*1rev24*.*2*^*/ATPsynC*^*2*^ trans-heterozygotes showed a significant decline in the locomotory performance compared to *ATPsynC*^*1rev18*.*3*^*/ATPsynC*^*2*^ and control flies Oregon-R and *ATPsynC*^*1rev17*.*1*^*/ATPsynC*^*2*^ (P < 0.01 Fig 3D, 3E and 3F).

Furthermore, mutant flies carrying these pleiotropic alleles display poor fertility. To dissect this phenotype, we set up a mating experiment where single virgin males and females from each pool of mutant (*ATPsynC*^*1rev18*.*3*^, *ATPsynC*^*1rev5*.*2*^ and *ATPsynC*^*1rev24*.*2*^) and control (*ATPsynC*^*1rev17*.*1*^) trans-heterozygotes were crossed separately with Oregon-R individuals to determine i) mating latency, ii) copulation duration, iii) egg production and iv) progeny production. In parallel, pure Oregon-R single crosses provided wild-type reference observations for the same traits.

**Fig 4:**
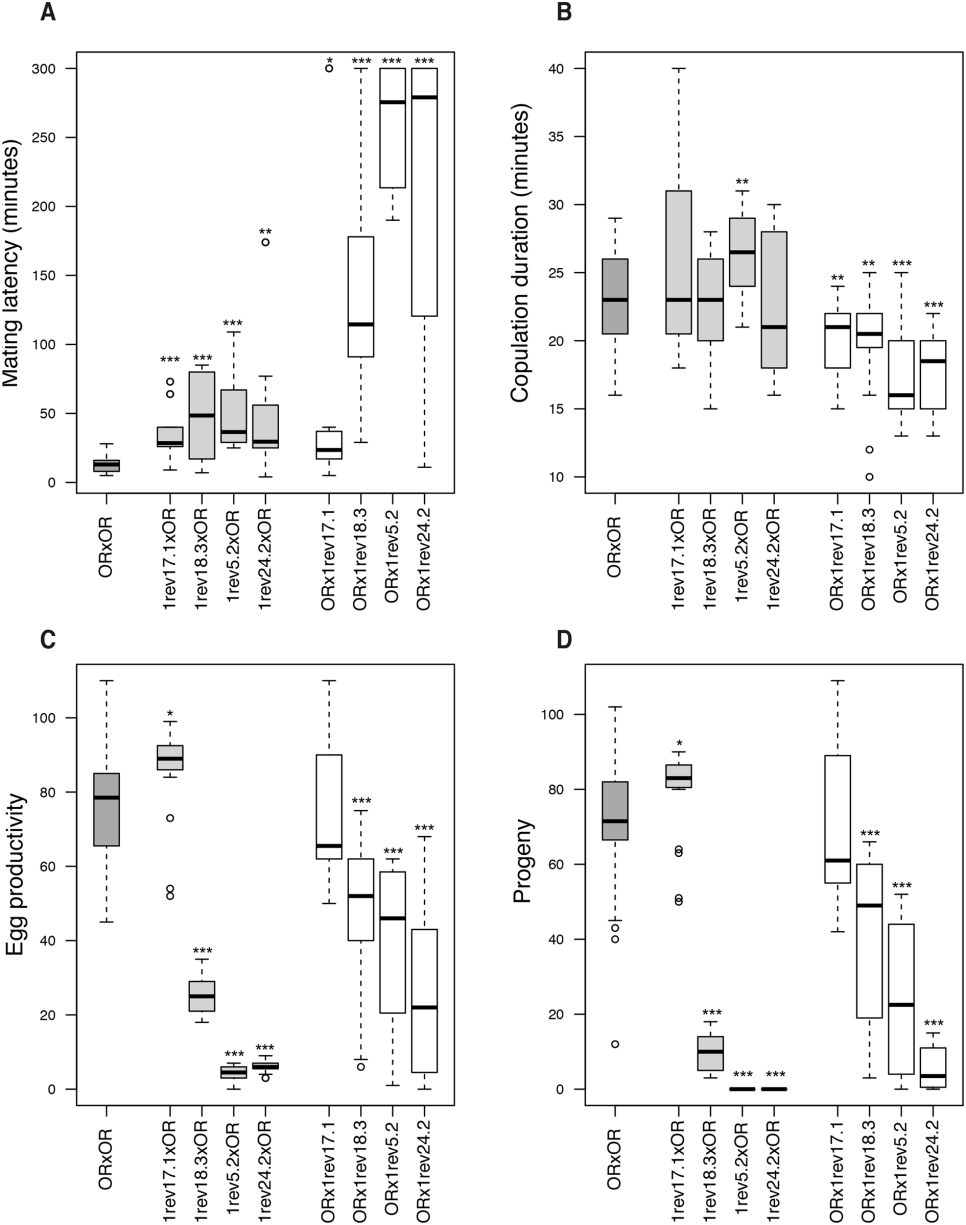
Mating and fertility analyses of trans-heterozygotes for *ATPsynC*^*2*^ revertant alleles. Mating latency (A), copulation duration (B), egg productivity (C) and progeny (D) in single crosses between wild-type Oregon-R (OR) flies and trans-heterozygotes for *ATPsynC*^*2*^ revertant alleles. By convention, each cross reads as female genotype X male genotype. “1revX.X” reads as *ATPsynC*^*1revX*.*X*^*/ATPsynC*^*2*^. Statistical significance codes (comparison to wild-type): *P < 0.05; **P < 0.01; ***P < 0.001.

We found that mating latency increases drastically in crosses where males *ATPsynC*^*1rev18*.*3*^*/ATPsynC*^*2*^, *ATPsynC*^*1rev5*.*2*^*/ATPsynC*^*2*^ and *ATPsynC*^*1rev24*.*2*^*/ATPsynC*^*2*^ take part (P < 0.001, Fig 4A). This is mostly due to erratic courtship behaviour of these males (see [31] for a review of the courtship behaviour in *Drosophila*), which are generally inactive, and even when they finally show interest in the female, it takes them several attempts before successfully starting copulation. Typically, during courtship, these males stop singing abruptly, lose or struggle to mount the female; otherwise the female rejects them by using a repelling behaviour (usually kicking). When copulation occurs, however, mutant males invest less time than control flies (P < 0.01, Fig 4B). Egg productivity is impaired in mutant females, but it is also less pronounced in wild-type Oregon-R females that have been mated with mutant males (P < 0.001, Fig 4C). As a result, progeny production is drastically affected (P < 0.001). In fact, *ATPsynC*^*1rev5*.*2*^*/ATPsynC*^*2*^ and *ATPsynC*^*1rev24*.*2*^*/ATPsynC*^*2*^ females are completely infertile, while *ATPsynC*^*1rev18*.*3*^*/ATPsynC*^*2*^ females and males of all the three mutant genotypes show different degrees of hypofertility (Fig 4D). The ANOVA test shows that *genotype* and *genotype*: *sex* interaction has the main effects on progeny production in our experiment (respectively: *F*_*4*,*151*_ = 160.864, *P* < 0.001; *F*_*5*,*151*_ = 11.058, *P* < 0.001), while *copulation duration*, for example, does not (*F*_*1*,*151*_ = 2.933, *P* > 0.05).

**Fig 5:**
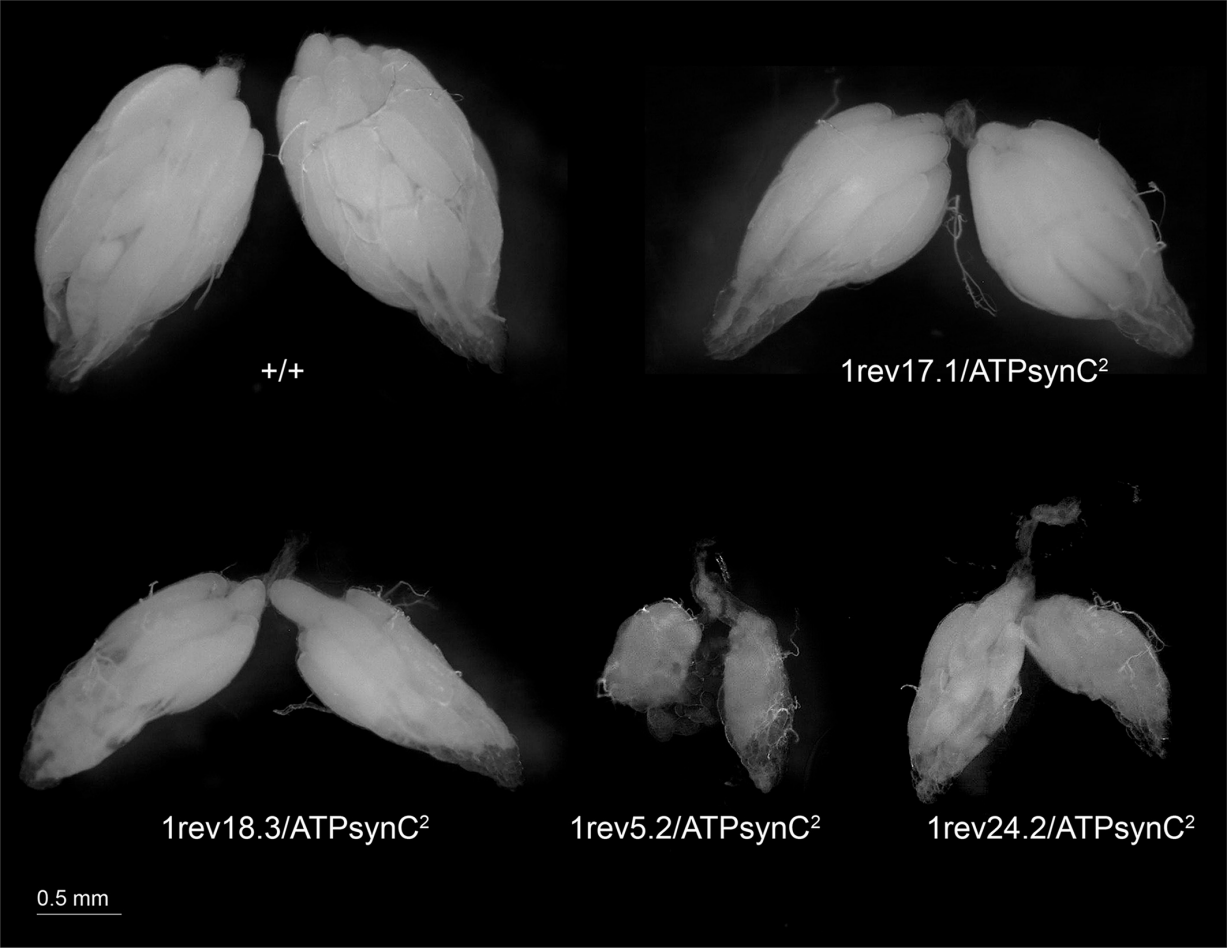
Aberrant gonadogenesis in females trans-heterozygotes for the *ATPsynC* pleiotropic mutations. Dissected ovaries from 5-days old females are compared. +/+ = Oregon-R female.

We subsequently investigated, primarily in females, if the infertility phenotype we observed was dependent on aberrant gonadogenesis. We dissected several adult, sexually mature females of the three mutant genotypes and found that their ovaries were atrophic and much smaller in size than wild-type Oregon-R and *ATPsynC*^*1rev17*.*1*^*/ATPsynC*^*2*^ control flies (Fig 5). Additionally, ovaries from *ATPsynC*^*1rev5*.*2*^*/ATPsynC*^*2*^ and *ATPsynC*^*1rev24*.*2*^*/ATPsynC*^*2*^ were usually smaller than *ATPsynC*^*1rev18*.*3*^*/ATPsynC*^*2*^ counterparts.

### Molecular characterization of *ATPsynC* mutant alleles

Given the broad range of phenotypes associated with mutations in *ATPsynC*, we investigated if the severity and the penetrance of those phenotypes were dependent on reduced levels of *ATPsynC* mRNA, ATP Synthase subunit c or ATP deficit in mutant flies.

We took advantage of the extraordinary conservation of the ATP synthase subunit c throughout long evolutionary times to test levels of protein expression in *ATPsynC* mutants by using a polyclonal antibody originally raised against the mature domain of the rabbit ATP synthase subunit c [32]. This antibody specifically recognises the mature *Drosophila* subunit c, which shows a migration pattern consistent with the predicted molecular weight of about 7 kDa.

**Fig 6:**
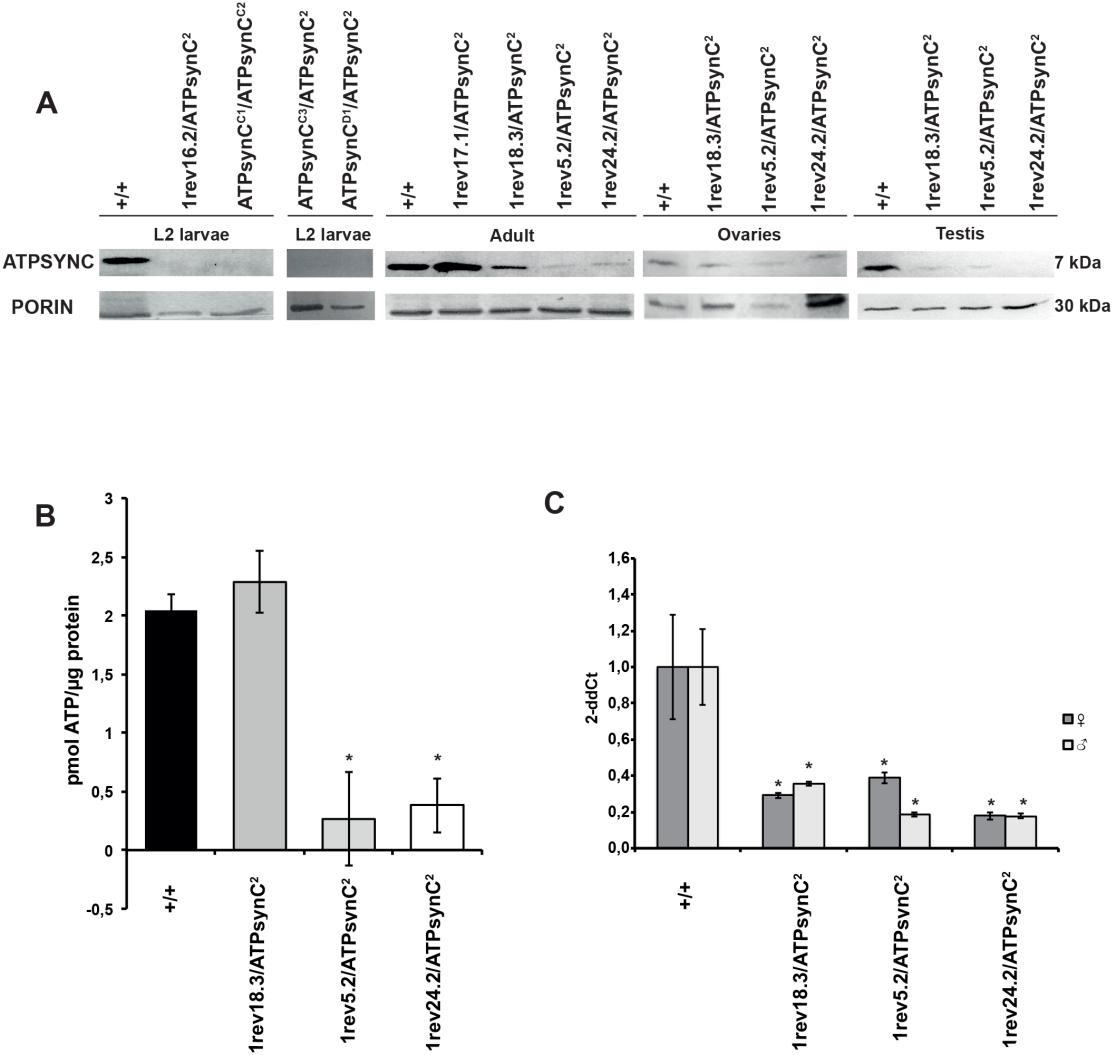
Molecular analyses of *ATPsynC* mutant alleles. (A) Western blot analyses of trans-heterozygotes for the *ATPsynC* mutations at critical stages and tissues during development. (B) ATP levels in whole-body lysates from control flies and trans-heterozygotes for the pleiotropic mutations of *ATPsynC*. (C) *ATPsynC* mRNA levels, measured by quantitative reverse transcription PCR (RT-qPCR), in control flies and trans-heterozygotes for the pleiotropic mutations of *ATPsynC*. Statistical significance codes (comparison to wild-type): *P < 0.05; **P < 0.01; ***P < 0.001. +/+ = Oregon-R flies.

While all the investigated mutant flies showed the presence of the mitochondrial marker Porin, which allows us to be confident about the mitochondrial content in our extracts, the amount of ATP synthase subunit c was found to be null or barely detectable in trans-heterozygotes for *ATPsynC* lethal alleles *ATPsynC*^*D1*^, *ATPsynC*^*C3*^, *ATPsynC*^*1rev16*.*2*^ and *ATPsynC*^*2*^, and significantly reduced in trans-heterozygotes for the *ATPsynC* pleiotropic alleles, with *ATPsynC*^*1rev18*.*3*^ displaying, however, more subunit c than *ATPsynC*^*1rev5*.*2*^ and *ATPsynC*^*1rev24*.*2*^ (Fig 6A and Table 1). Wild-type revertant allele *ATPsynC*^*1rev17*.*1*^, instead, showed a similar amount of subunit c compared to control Oregon-R flies (Fig 6A and Table 1). The same type of analysis, conducted on ovaries and testis dissected from trans-heterozygotes for alleles *ATPsynC*^*1rev18*.*3*^, *ATPsynC*^*1rev5*.*2*^ and *ATPsynC*^*1rev24*.*2*^, also showed a decreased level of subunit c in gonads from mutant flies (Fig 6A), suggesting that these mutations affect *ATPsynC* expression ubiquitously.

As the bulk of cellular ATP truly depends on the subunit c function, we asked if the change in the level of the subunit c protein, observed by western blot in the mutant flies, could interfere with the cellular energetic production. We measured the total ATP levels in whole-body lysates from control Oregon-R flies and mutant trans-heterozygotes for *ATPsynC*^*1rev18*.*3*^, *ATPsynC*^*1rev5*.*2*^ and *ATPsynC*^*1rev24*.*2*^ alleles. As shown in Fig 6B, we found a strong decrease in ATP levels in mutants *ATPsynC*^*1rev5*.*2*^ and *ATPsynC*^*1rev24*.*2*^ but not in *ATPsynC*^*1rev18*.*3*^ when compared to control Oregon-R flies, as expected considering the penetrance of these mutations.

Finally, we investigated the levels of cellular *ATPsynC* mRNA in the pleiotropic mutants. To overcome the limited sensitivity of the Northern blot hybridization, which did not allow detection of the ATPsynC transcripts in mutant strains (not shown), we performed qRT-PCR analyses and found a strong mRNA decrease in both mutant adult males and females compared to Oregon-R individuals (P < 0.05, Fig 6C). Interestingly, while *ATPsynC1rev*^*18*.*3*^ essentially shows the same amount of transcripts of *ATPsynC1rev*^*5*.*2*^ and *ATPsynC1rev*^*24*.*2*^, its protein level is higher, indicating a major RNA stability or translation efficiency. Furthermore, the level of protein in the *ATPsynC1rev*^*18*.*3*^ is enough to ensure a normal total ATP level (as Oregon-R), suggesting a threshold of ATPsynC protein content to ensure ATP synthase activity.

Overall, these results indicate that lethal alleles of *ATPsynC* block the development of flies during larval stages because they do not express adequate amounts of functional subunit c required for the correct activity of the mitochondrial ATP synthase. This hypothesis is also well supported by the fact that *ATPsynC* is highly expressed during first-, second- and early third-instar larval stages, which correspond exactly to the developmental points where *ATPsynC* lethal mutations manifest their effects.

On the other hand, pleiotropic alleles *ATPsynC*^*1rev18*.*3*^, *ATPsynC*^*1rev5*.*2*^ and *ATPsynC*^*1rev24*.*2*^ proved to be hypomorphic for ATP Synthase subunit c expression.

It has been show that ATP synthase dimers are involved in the control of biogenesis of the inner mitochondrial membrane [33].

**Fig 7:**
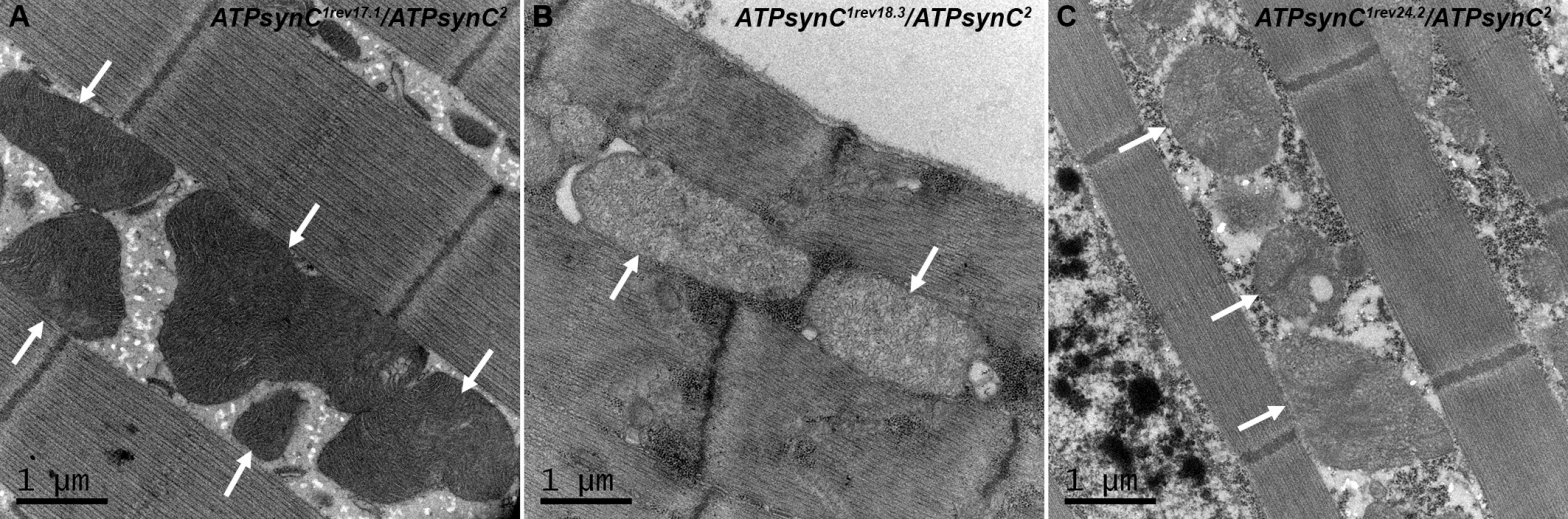
Mitochondria morphology analysis in trans-heterozygotes for representative *ATPsynC*^*1*^ revertant alleles. Mitochondria in thoracic flight muscles were examined in adult trans-heterozygotes for representative alleles *ATPsynC*^*1rev17*.*1*^ (A), *ATPsynC*^*1rev18*.*3*^ (B) and *ATPsynC*^*1rev24*.*2*^ (C) by using TEM. Arrows point to mitochondria.

Therefore, in order to investigate if the reduced expression of the subunit c protein resulted in altered mitochondrial morphology, we used TEM to examine mitochondria in thoracic flight muscles of adult trans-heterozygotes carrying the representative alleles *ATPsynC*^*1rev18*.*3*^ and *ATPsynC*^*1rev24*.*2*^ and compared these findings to mitochondria from wild-type trans-heterozygotes for the allele *ATPsynC*^*1rev17*.*1*^. We found a strong decrease in the overall electron density of the mitochondria, indicative of poor functionality (Fig 7). Moreover, the inner mitochondrial membrane folds (cristae) have reduced density in mutant flies if compared to control. The *ATPsynC*^*1rev24*.*2*^ mutant also showed a pronounced vacuolization. Since these structures determine the assembly and stability of respiratory chain complexes and hence mitochondrial respiratory efficiency [34], we confirmed the correlation between a reduced amount of functional subunit c, an alteration of cristae, and a bioenergetic deficit.

To analyse the mitochondrial network in thoracic muscles, we constructed recombinant flies carrying a mitochondrial-targeted YFP protein (mito-YFP) in *cis* of insertion *ATPsynC*^*1*^ and generated trans-heterozygote flies for mutant alleles *ATPsynC*^*1rev18*.*3*^, *ATPsynC*^*1rev24*.*2*^ and wild-type *ATPsynC*^*1rev17*.*1*^.

**Fig 8:**
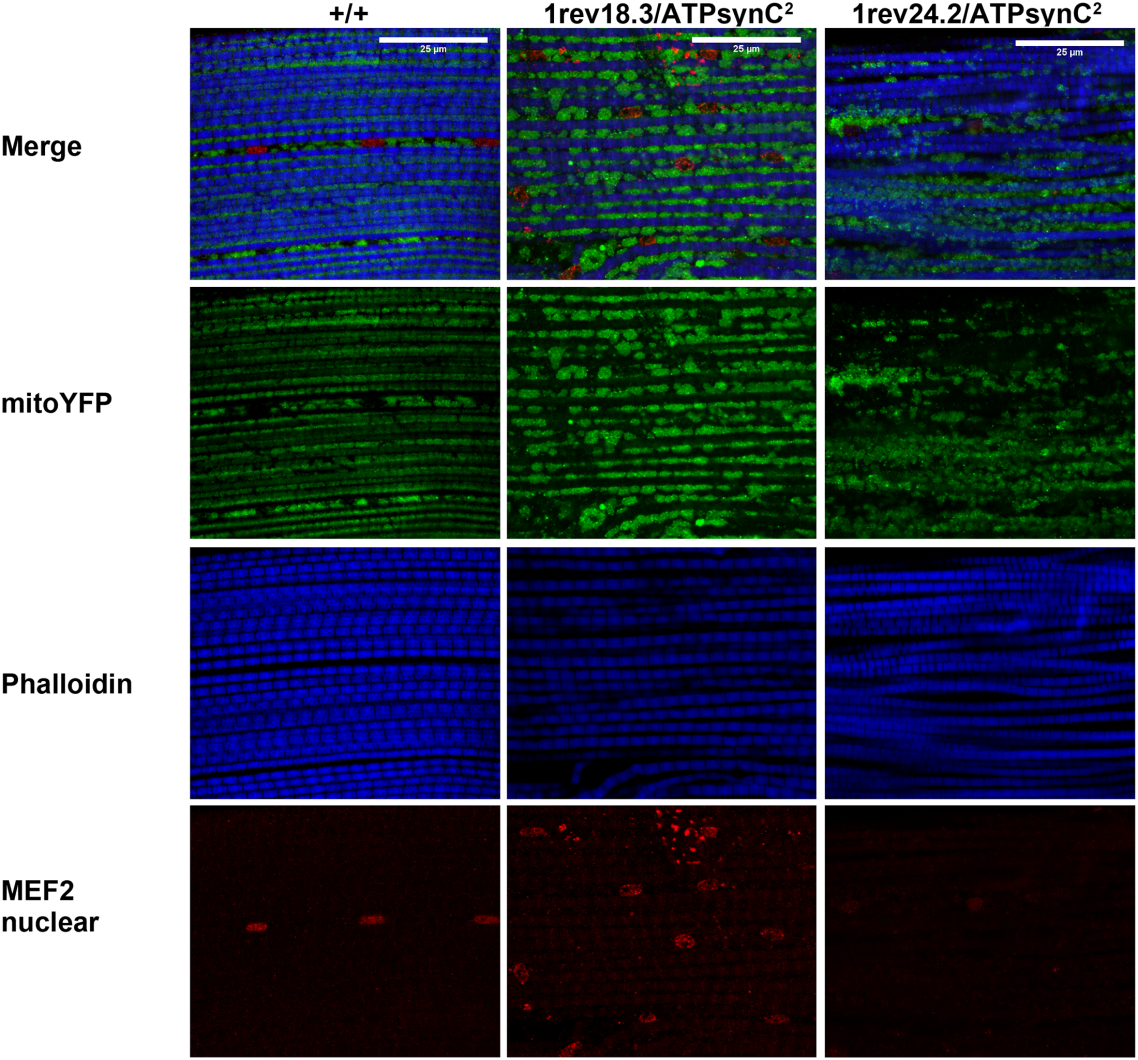
Mitochondrial ultrastructure in trans-heterozygotes for representative *ATPsynC*^*1*^ revertant alleles. Thoracic muscles from Oregon-R wild-type flies plus mutant flies *ATPsynC*^*1rev18*.3^ and *ATPsynC*^*1rev24*.2^, stained with an anti-GFP antibody selective for mitochondria in green, an anti-phalloidin antibody to label myofibrils in blue, and an anti MEF2- antibody for nuclear localization in red. Oregon-R flies showed a tubular mitochondrial networks that enclose myofibrils with big aligned nuclei. Mitochondria are bigger and clumping with a slight alteration of mitochondrial network in *ATPsynC*^*1rev18*.3^ mutants, whereas in *ATPsynC*^*1rev*24.2^ flies mitochondria are smaller, clumped and badly organized around myofibrils.

As shown in Fig 8, thoracic muscles in wild-type flies consist of well-organized, tubular and evenly distributed fibres, where myofibrils are enclosed by mitochondria. In contrast, thoracic muscle sections of *ATPsynC*^*1rev18*.*3*^ mutant flies show molecular alterations of the mitochondrial network, with many large mitochondrial clumps. However a residual healthy mitochondrial network is still preserved. In preparations from *ATPsynC*^*1rev18*.*3*^ flies, we also observed an increase in mitochondrial size that could be putatively related to an attempted increase in mitochondrial biogenesis to compensate the energetic deficit (like red ragged fibres in mitochondrial related diseases) [35, 36]. On the contrary, we observed a decrease in mitochondrial size in thoracic muscle preparations from *ATPsynC*^*1rev24*.*2*^ mutant flies, where prominent mitochondrial fragmentation and clumping are easily detectable, resulting in a severe alteration of the mitochondrial network.

Interestingly, these data tightly correlate with the overall severity and penetrance of our pleiotropic *ATPsynC* alleles, indicating a robust structure-function relationship. Taken together, our structural observations suggest that *ATPsynC* is an essential gene involved in the regulation of mitochondrial morphology and networking, by regulating ATP synthase stability.

## Discussion

For aerobic organisms, OXPHOS is central to life. All the enzymes involved must interact and work harmoniously to efficiently produce energy to power up the cell. For this reason, OXPHOS components tend to be highly conserved throughout evolution [28] and mutations affecting their function and/or stoichiometry are extremely deleterious, usually incompatible with life [37].

In humans, inborn deficiencies of mitochondrial ATP synthase, the terminal actor of the OXPHOS pathway, cause severe neuromuscular disorders. All patients identified so far presented neonatal onset and more than one half of them died within the first week of life, with surviving patients showing multiple motor and cognitive defects [12].

The use of animal models has allowed great advances in the understanding of the pathophysiology of diseases related to mitochondrial defects [38]. However, little success has been achieved in generating a comprehensive genetic model for mitochondrial ATP synthase disorders.

In *D*. *melanogaster* a single mutation in the mitochondrial gene *ATP6* causes adult-onset neuromuscular dysfunction and myodegeneration [16], while mutations in nuclear genes *bellwether* and *stunted*, encoding the ATP synthase subunits *α* and *ε*, cause lethal and male sterile phenotypes, respectively [39–41]. A recent knock-down analysis of the *ATPsyn-b* gene, using an RNAi transgenic construct, also shows an essential role of this gene in fly viability and male fertility [42].

In this paper, we targeted *ATPsynC*, the *D*. *melanogaster* gene encoding for the subunit c of the mitochondrial ATP synthase, to produce a comprehensive genetic model based on a large set of mutations showing phenotypes ranging from lethality to complex pleiotropy.

Using EMS mutagenesis, we generated two independent missense mutations in the coding sequence of *ATPsynC*. Interestingly, although these mutations affect different portions of the premature polypeptide, P103L in the mature domain and S59L in an evolutionary conserved region of the putative mitochondrial import peptide (Fig 1), we found that flies carrying these substitutions lack the ATP synthase subunit c and do not develop further than the first-instar larval stage. While P103L missense is likely to impair the correct functionality of the mature subunit c, S59L could abolish its correct maturation since it is located very close to the putative cleavage site. However, in both cases we do not see an accumulation of the mutated subunit c, indicating that these protein variants are promptly degraded.

In parallel, we took advantage of the existence of two *D*. *melanogaster* lines carrying a P-element insertion in the 5’UTR of *ATPsynC*, in positions also close to the TSS, to generate mutations that altered the expression of *ATPsynC*. Such mutations were predicted to be highly deleterious, given the fact that the subunit c is the most expressed protein of the OXPHOS system, although this may not be universally true, as for the case of the mammalian brown adipose tissue [43]. To this end, we isolated and characterized mutations caused by imprecise P-element excision events (see Results).

**Fig 9:**
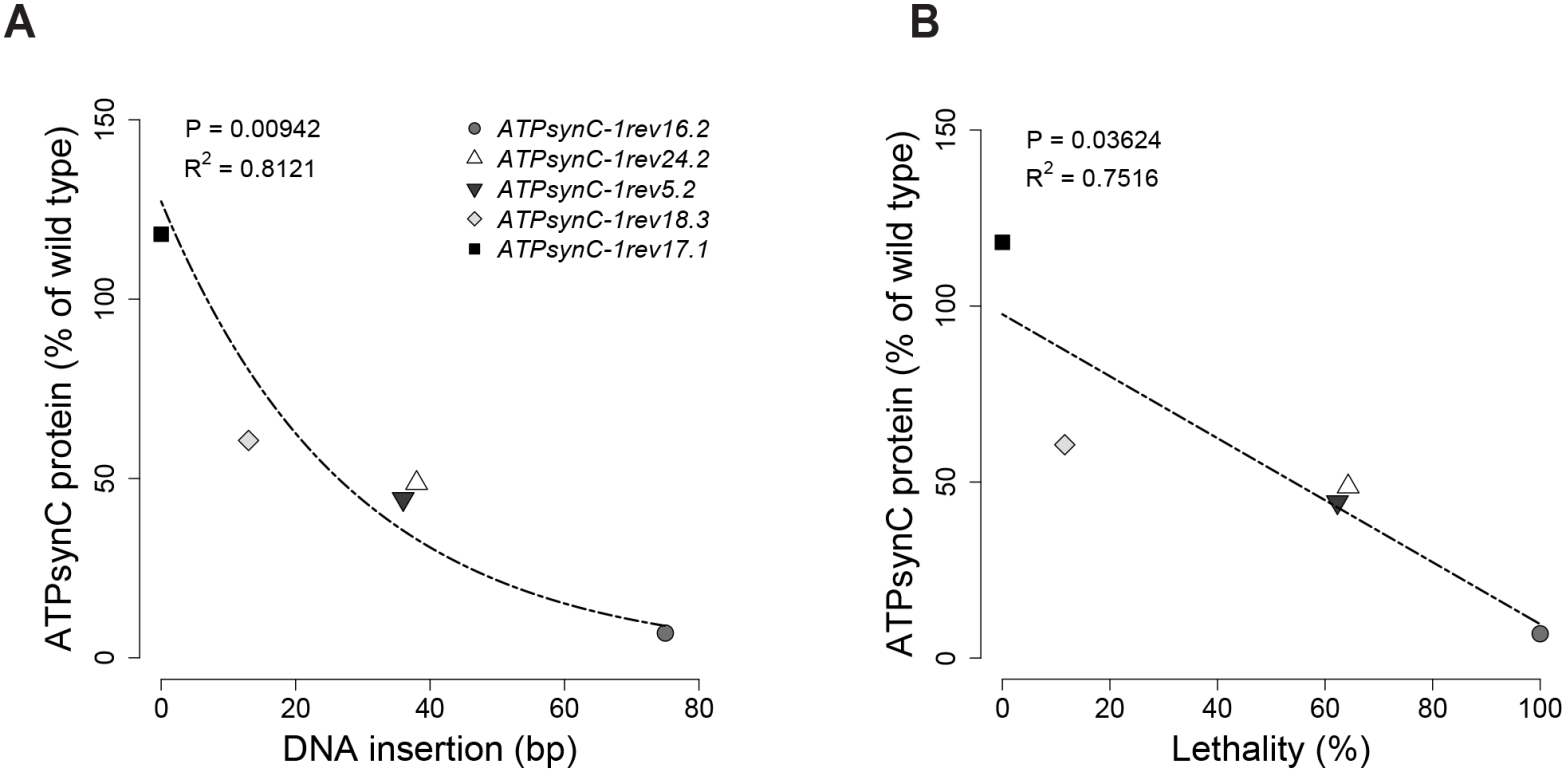
The *ATPsynC* model. Relations between cellular levels of mitochondrial ATP synthase subunit c and: (A) the amount of the extra DNA left at the excision site, expressed in base pairs (bp); (B) lethality in trans-heterozygotes for *ATPsynC*^*1*^ revertant alleles.

A particular set of such mutations—*ATPsynC*^*1rev18*.*3*^, *ATPsynC*^*1rev5*.*2*^, *ATPsynC*^*1rev24*.*2*^ and *ATPsynC*^*1rev16*.*2*^, respectively carrying an extra DNA fragment of 13, 36, 38, and 75 base pairs inserted at the same nucleotide position within the 5’UTR of *ATPsynC* (Fig 2C), provided us an ideal toolkit to test *in vivo* consequences of decreasing subunit c expression (Table 1). In fact, we found a strong negative correlation between the level of cellular subunit c and the amount of extra DNA left at the excision site (Fig 9A) and with the penetrance of the pathological condition (Fig 9B).

We confirmed, by qRT-PCR analyses, that the imprecise P-element excisions cause a strong decrease in the cellular content of *ATPsynC* mRNA in alleles *ATPsynC*^*1rev18*.*3*^, *ATPsynC*^*1rev5*.*2*^ and *ATPsynC*^*1rev24*.*2*^. Although we did not investigate in further details the molecular mechanism at the basis of this evidence, we hypothesise that these mutations may affect the stability of the nascent pre-mRNA and/or the mature mRNA. In fact, by simulating the 5’UTR secondary structures of all these alleles, we found that in all the cases the residual P-element DNA fragment left after the excision was capable of forming a strong stem-loop structure by employing complementary sequences left at the excision site, corresponding to portions of the inverted terminal repeats of the P-element (S3 Fig). Alternatively, these exogenous sequences may interfere with the correct positioning of transcription factors and/or the RNA polymerase II at the *ATPsynC* promoter.

Given the strong decrease of *ATPsynC* mRNA in alleles *ATPsynC*^*1rev18*.*3*^, *ATPsynC*^*1rev5*.*2*^ and *ATPsynC*^*1rev24*.*2*^ (Fig 6C), and the overall extreme consequences of the original P-element insertions and their derived lethal alleles, we also argue that the main transcriptional unit at the *ATPsynC* locus corresponds to *ATPsynC-RA* (FlyBase ID FBtr0085772). We found support for this hypothesis by analysing the FlyBase Genome Browser [44, 45] for uniquely-mapped modENCODE RNA-seq reads for *ATPsynC* (S4 Fig). Also, as suggested by the annotated cDNA sequences (S4 Fig), this main transcriptional unit is responsible for the generation of two mRNA isoforms, which differ only for their transcription termination sites within the terminal exon, and which are likely to correspond to the two only transcripts identified by our Northern blot analysis. Other annotated *ATPsynC* transcripts may represent cryptic isoforms that are probably expressed in specific cell types and temporal windows, with still unclear roles.

The complex pleiotropic phenotype associated with alleles *ATPsynC*^*1rev18*.*3*^, *ATPsynC*^*1rev5*.*2*^ and *ATPsynC*^*1rev24*.*2*^ is likely attributable to strong alterations of the mitochondrial molecular structure and distribution (Figs 7 and 8) and to the consequent drastic depletion in ubiquitous energy production (Fig 6B). This is especially evident for alleles *ATPsynC*^*1rev5*.*2*^ and *ATPsynC*^*1rev24*.*2*^, which show an extreme penetrance of the phenotype. To our knowledge, these mutations are among the most pleiotropic mutations ever reported in *Drosophila* and this is in line with expectations for highly expressed genes encoding slowly evolving proteins [46], such as *ATPsynC*.

Interestingly, the lethal phenotype that we observe at transitions to adult stage for alleles *ATPsynC*^*1rev18*.*3*^, *ATPsynC*^*1rev5*.*2*^ and *ATPsynC*^*1rev24*.*2*^ (Fig 3C) largely recapitulate lethality observed in human newborn patients with genetic defects in mitochondrial ATP synthase, as 50% or more of the patients die within the first week of life, while the survivors show complex, severe disorders. This patho-phenotype also suggests that the genetic background plays an important role in the onset and the penetrance of the pathology via epistatic interactions, as exemplified by the pathological spectrum of patients homozygous for the c.317-2A>G mutation in *TMEM70* [47, 48]. We therefore believe that alleles *ATPsynC*^*1rev18*.*3*^, *ATPsynC*^*1rev5*.*2*^ and *ATPsynC*^*1rev24*.*2*^ represent a useful tool to identify evolutionary conserved genetic interactions at the basis of such complex disorders by screening for genetic suppressors and enhancers in our model system. Putative modifiers may, in fact, suggest new possible therapeutic solutions.

Furthermore, since allele *ATPsynC*^*1rev16*.*2*^ and other null mutations, produced in this work, all manifested pre-adult, early life lethality (see Table 1), our animal model indicates an overall scenario where, depending on the strength of the mutation, there are three broad outcomes from mitochondrial ATP synthase aberrations in *Drosophila*: i) pre-adult lethality; ii) multi-trait pathology accompanied by early adult lethality; iii) multi-trait adult pathology. This speculation is also supported by the fact that all the nuclear genes encoding subunits of the mitochondrial ATP synthase show a striking coordination of their temporal expression profile, with peaks of expression during early larval stages and at transition from pupal to adult stages (Fig 2D).

In mammals, the expression of ATP synthase genes is high since the embryonic stage and mouse mutants for *ATP5A1*, *ATP12* and *ATP5B* genes show embryonic and pre-weaning lethality (consult http://www.informatics.jax.org/ for expression data and phenotypes associated with nuclear genes encoding mitochondrial ATP synthase subunits in mouse). Reasonably, the pathological spectrum predicted in *D*. *melanogaster* may be paralleled in humans too, where mutations affecting the mitochondrial ATP synthase would generally result in 1) embryonic lethality, 2) neonatal lethality and 3) postnatal/adulthood severe pathology. Considering that genes encoding ATP synthase subunits and chaperones are essential for life, since they seat at the very foundations of cellular energy metabolism, and that human pathologies arising from mutations in these gene could be underestimated in number, our model might be useful for future studies, aimed at understanding the role of ATP synthase subunits in human mitochondrial related diseases.

## Supporting information

S1 Fig Developmental expression of *ATPsynC* in *Drosophila*. (A) Expression pattern of *ATPsynC* during development, obtained from modENCODE data. (B) RT-qPCR analysis of *ATPsynC* expression in whole bodies of 5 days old adult males and females.
(TIF)

S2 Fig P-element insertions within *ATPsynC*. Annotated screenshot from the UCSC genome browser where the locations of the P-elements inserted in the 5’UTR of *ATPsynC* are highlighted in relation to the local sequence conservation.
(TIF)

S3 Fig Sequence and secondary structure comparisons of principal 1rev alleles of *ATPsynC*. (A) Multiple alignment of alleles *ATPsynC*^*1rev18*.*3*^, *ATPsynC*^*1rev5*.*2*^, *ATPsynC*^*1rev24*.*2*^ and *ATPsynC*^*1rev16*.*2*^. (B) Secondary structure comparison of nascent *ATPsynC* RNAs from wild-type *ATPsynC* (i), *ATPsynC*^*1rev18*.*3*^ (ii), *ATPsynC*^*1rev5*.*2*^ (iii) *ATPsynC*^*1rev24*.*2*^ (iv) and *ATPsynC*^*1rev16*.*2*^ (v).
(TIF)

S4 Fig Expression associated to *ATPsynC* transcriptional units. Uniquely mapping RNAseq data from the modENCODE project elucidate main expression at the *ATPsynC* locus: *ATPsynC-RA* being the predominant transcriptional unit and generating two polyadenylated mRNA forms.
(TIF)
