# Supplementary figures and images for: Characterization of Drosophila ATPsynC mutants as a new model of mitochondrial ATP synthase disorders
Source: PLoS One. 2018 Aug 10;13(8):e0201811. doi: 10.1371/journal.pone.0201811 (PMC6086398; doi:10.1371/journal.pone.0201811)

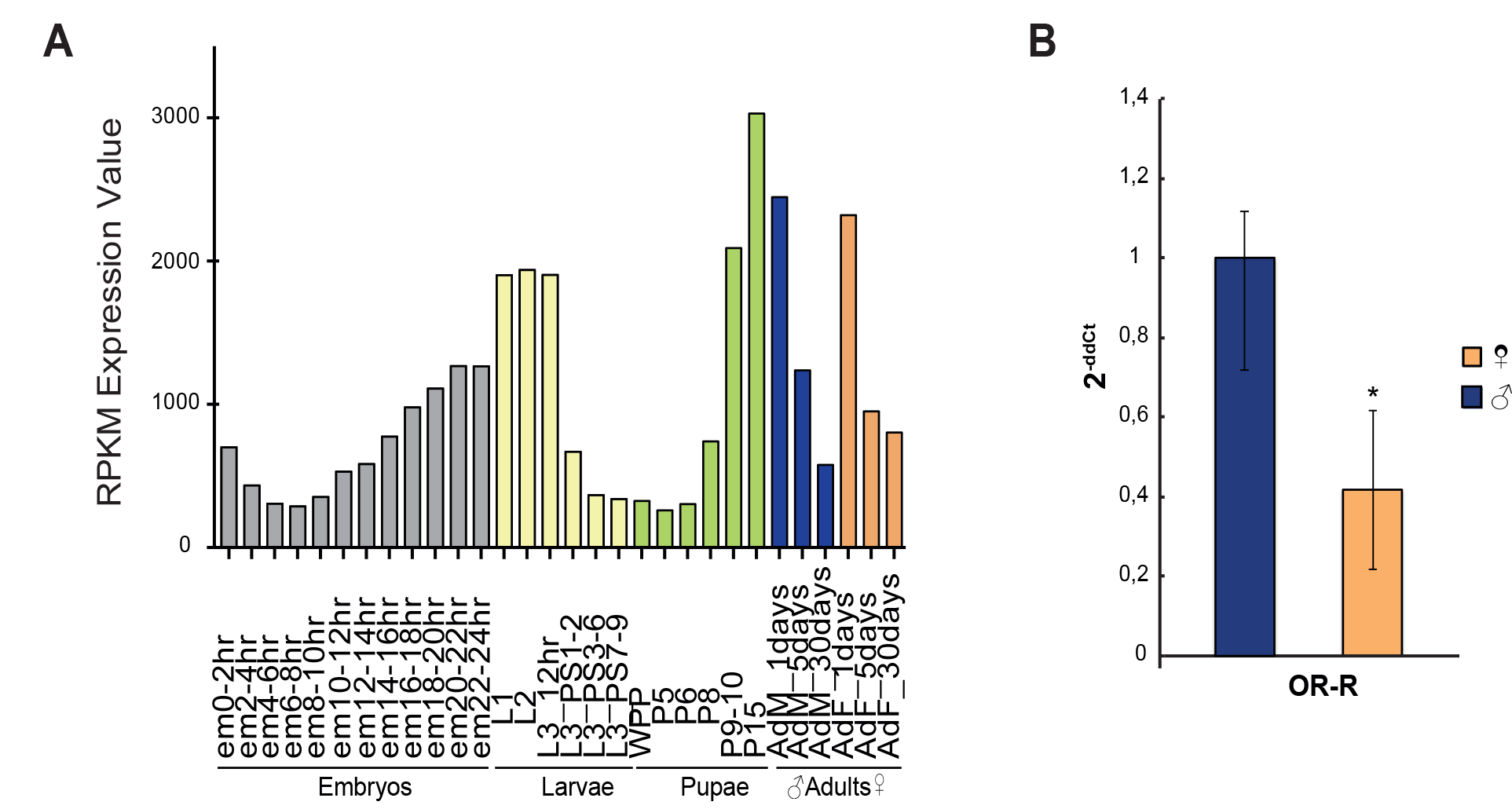

Supplement: S1 Fig — (A) Expression pattern of ATPsynC during development, obtained from modENCODE data. (B) RT-qPCR analysis of ATPsynC expression in whole bodies of 5 days old adult males and females. (TIF) [file pone.0201811.s001.tif]

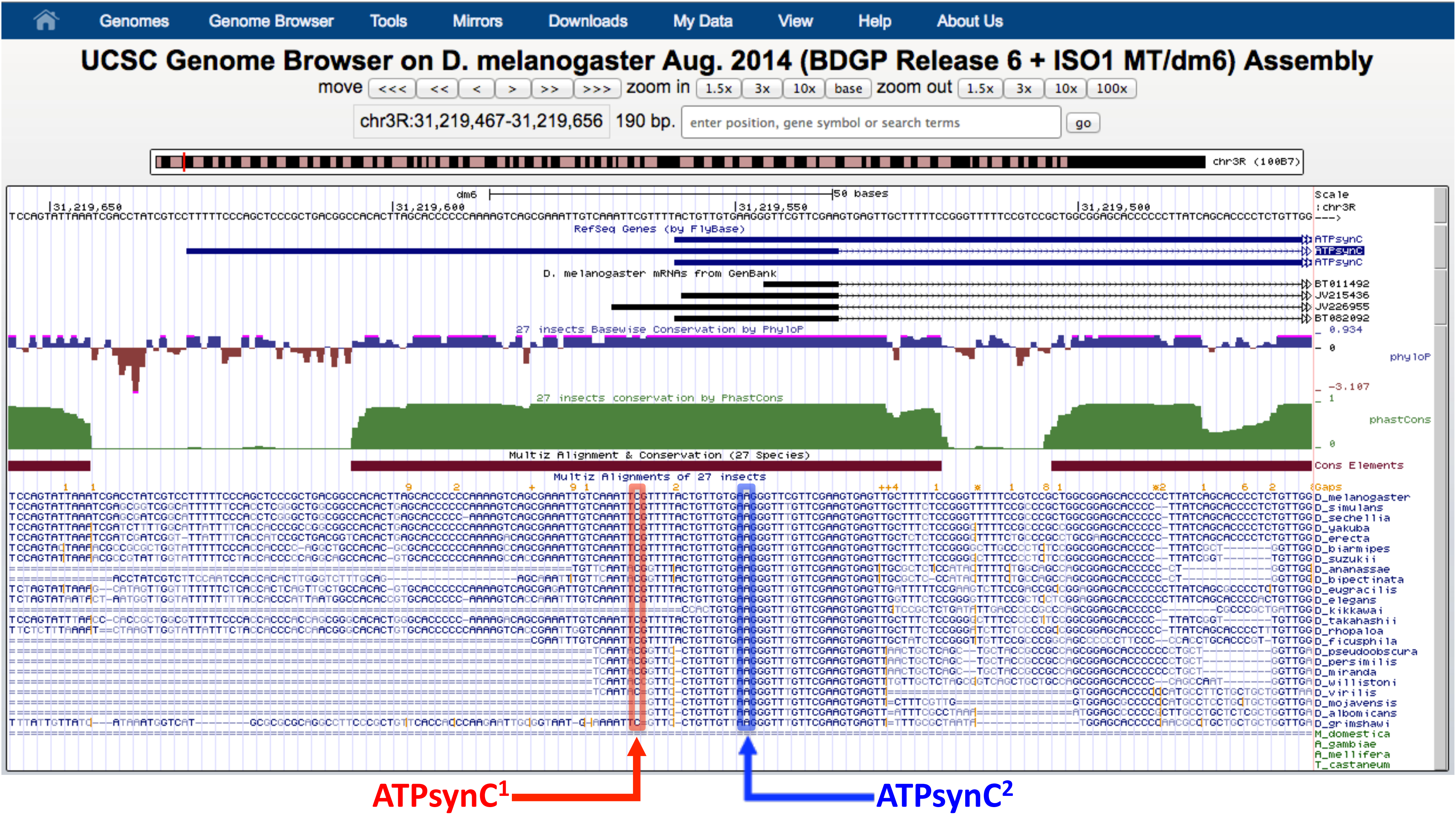

Supplement: S2 Fig — Annotated screenshot from the UCSC genome browser where the locations of the P-elements inserted in the 5’UTR of ATPsynC are highlighted in relation to the local sequence conservation. (TIF) [file pone.0201811.s002.tif]

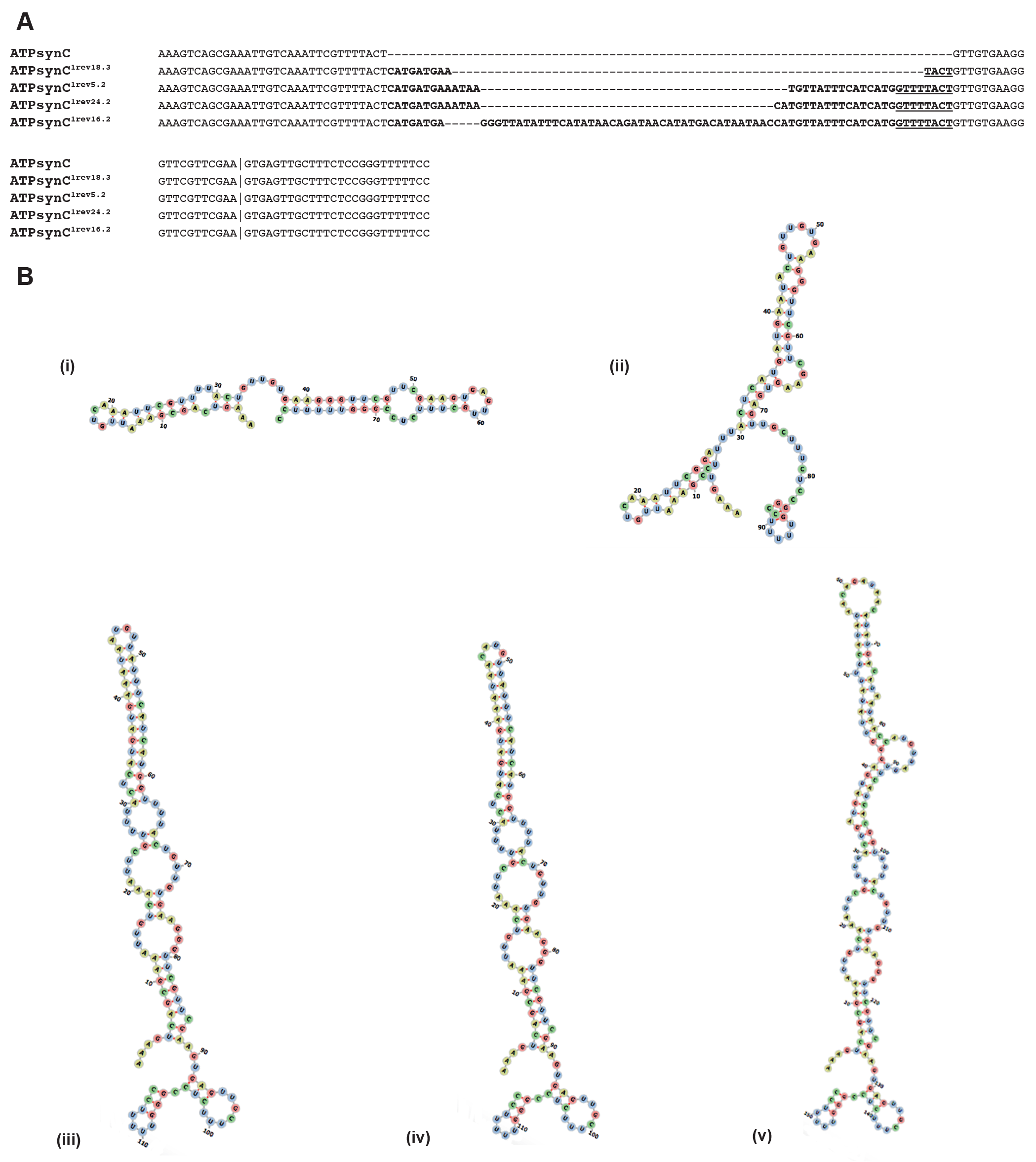

Supplement: S3 Fig — (A) Multiple alignment of alleles ATPsynC1rev18.3, ATPsynC1rev5.2, ATPsynC1rev24.2 and ATPsynC1rev16.2. (B) Secondary structure comparison of nascent ATPsynC RNAs from wild-type ATPsynC (i), ATPsynC1rev18.3 (ii), ATPsynC1rev5.2 (iii) ATPsynC1rev24.2 (iv) and ATPsynC1rev16.2 (v). (TIF) [file pone.0201811.s003.tif]

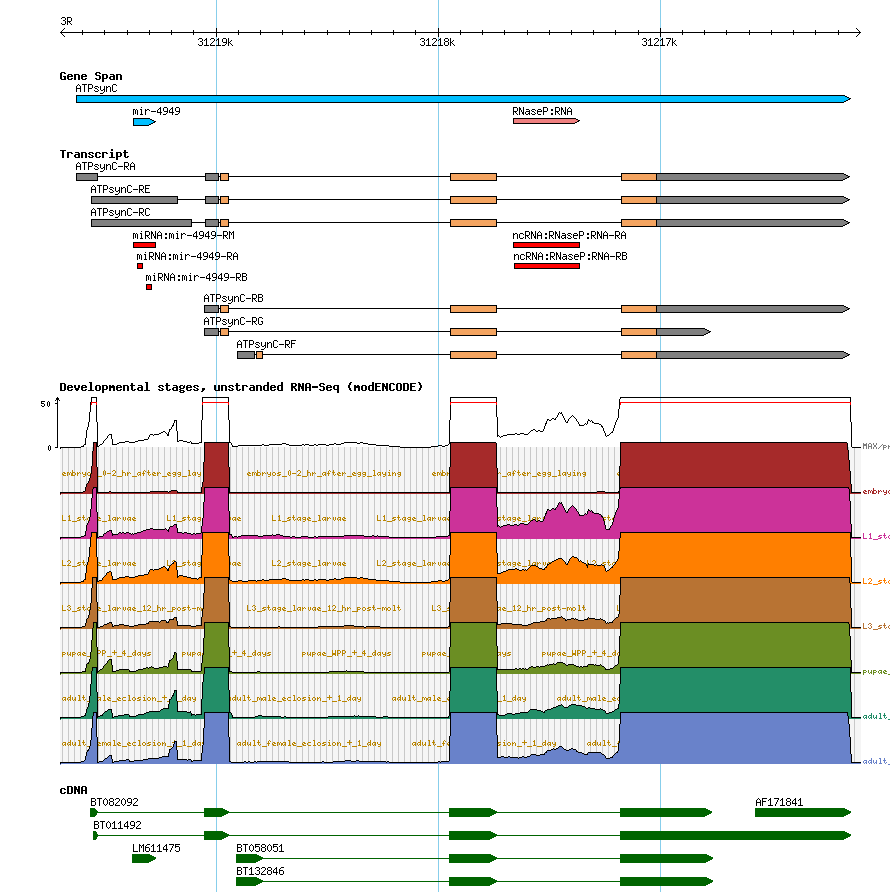

Supplement: S4 Fig — Uniquely mapping RNAseq data from the modENCODE project elucidate main expression at the ATPsynC locus: ATPsynC-RA being the predominant transcriptional unit and generating two polyadenylated mRNA forms. (TIF) [file pone.0201811.s004.tif]
